# Male-based key to genera for the subfamily Formicinae (Hymenoptera, Formicidae) in the Nearctic region

**DOI:** 10.3897/zookeys.1280.176598

**Published:** 2026-05-20

**Authors:** Nicole Rasoamanana, Joe A. MacGown, Brian L. Fisher, JoVonn G. Hill

**Affiliations:** 1 Department of Agricultural Science and Plant Protection, Mississippi State University, Starkville, MS 39762, USA California Academy of Sciences San Francisco United States of America; 2 California Academy of Sciences, San Francisco, CA 94118, USA Department of Agricultural Science and Plant Protection, Mississippi State University Starkville United States of America

**Keywords:** Biodiversity surveys, dichotomous key, male genitalia, systematics, wing venation

## Abstract

Male ants remain one of the least documented sources of morphological information in ant taxonomy, despite their frequent occurrence in biodiversity surveys and their potential value for systematic research. Within the ant subfamily Formicinae, identification resources for male ants are particularly limited in the Nearctic region. The only comprehensive treatment remains Smith’s (1943) key to male ants of the United States, which predates modern taxonomic revisions and advances in morphological terminology. In this work, a revised identification key to the genera of Nearctic Formicinae is presented, based exclusively on male morphology. An illustrated key to the 14 formicine genera recorded from the Nearctic region is provided, supported by updated diagnoses and figures of diagnostic characters, including genitalia and wing venation.

This study provides the first modern synthesis of male morphological characters for Nearctic Formicinae and enables reliable identification of males, including specimens collected independently of workers in light traps, Malaise traps, and flight-intercept traps, thereby facilitating the broader integration of male morphology into taxonomic and biodiversity research on ants.

## Introduction

The subfamily Formicinae is a widely distributed and species-rich lineage of ants characterized by broad ecological adaptability and occurrence in a wide range of habitats worldwide. It comprises 55 valid genera and 3287 valid species (Bolton 2025), making it one of the largest subfamilies within Formicidae. Members of Formicinae are ecologically dominant in many terrestrial ecosystems and exhibit diverse life histories, nesting behaviors, and foraging strategies. In the Nearctic region, the subfamily is represented by 14 genera and 529 species (AntWeb 2025), reflecting a substantial portion of regional ant diversity.

Historically, ant taxonomy has focused heavily on the worker and queen castes, resulting in a disproportionately low number of male-based descriptions. The male caste remains undocumented for many taxa, particularly in faunal inventories and revisionary works. This underrepresentation limits species-level taxonomy as well as comparative analyses of morphological evolution, reproductive strategies, and phylogenetic inference. Identifying male ants to genus or species level is especially challenging when they are collected in isolation, such as in light trap surveys, flight intercept traps, or Malaise traps, where no associated workers are available. Despite these difficulties, males often exhibit distinctive morphological characters, particularly in genitalia, wing venation, and head structure, which are highly informative for generic and species-level discrimination. Advances in molecular phylogenomics, particularly the use of ultra-conserved elements (UCEs) combined with detailed morphological analyses, have significantly improved our understanding of evolutionary relationships within Formicinae (Blaimer et al. 2015; Ward and Boudinot 2021). These integrative approaches underscore the importance of including all castes, especially males, in systematic studies to achieve more robust and complete phylogenetic frameworks. Notably, recent work has begun to explicitly integrate UCE-based phylogenomic data with detailed analyses of male morphology (e.g., Williams et al. 2024), demonstrating that male characters provide important complementary evidence for interpreting phylogenetic relationships.

In recent years, the taxonomic value of male morphology has been increasingly recognized. Studies such as Boudinot (2015), Cantone (2017), and Ramamonjisoa et al. (2024) have highlighted the importance of the male caste in resolving taxonomic ambiguities and uncovering hidden diversity. Furthermore, a growing number of revisionary works have begun to systematically describe males alongside workers and queens, and to incorporate male-based identification keys at both genus and species levels (LaPolla 2004; Messer et al. 2016). As part of ongoing taxonomic efforts in the Nearctic region, the present study addresses a long-standing gap by providing a revised, morphology-based identification key to the genera of Nearctic Formicinae, based solely on male characters. This key builds upon early work by Smith (1943), who published a key to male ants of the United States and aims to improve the accuracy of genus-level identification for male specimens, including those collected independently of workers, and to facilitate their broader inclusion in taxonomic, ecological, and phylogenetic research. This study provides the first modern, male-based identification framework for Nearctic Formicinae since Smith (1943), filling a longstanding gap and promoting a more complete and balanced understanding of ant diversity, systematics, and evolution.

## Materials and methods

### Taxonomic framework

Generic classification and taxonomic concepts for Nearctic Formicinae follow recent phylogenetic and systematic studies (Blaimer et al. 2015; Ward and Boudinot 2021) and the global ant catalog AntCat (Bolton 2025). Generic composition of the Nearctic fauna follows AntWeb (2025) and associated specimen records. Distributional information for each genus was compiled primarily from specimen records available on AntWeb (antweb.org) and supplemented with occurrence data from AntMaps (antmaps.org).

### Morphological terminology

Morphological terminology used in this study follows established references in ant taxonomy. Terminology for setae and setation follows Wilson (1955); wing venation follows Yoshimura and Fisher (2009); male genitalia follows Boudinot (2013), although traditional terms (e.g., cuspis) are retained where appropriate to maintain consistency with the broader ant taxonomic literature; and terminology for head and mesosoma morphology follows Yoshimura and Fisher (2007) and Boudinot and Fisher (2013). Terminology is applied consistently throughout the manuscript to facilitate comparison with previous taxonomic studies.

### Examined material

Specimens examined in this study are deposited in multiple institutional and private collections (see Depositories), with the majority of material housed in the Mississippi Entomological Museum (**MEM**), Starkville, Mississippi, USA (>1500 pinned specimens), and the California Academy of Sciences (**CASC**), San Francisco, California, USA. Where available, multiple specimens representing several species per genus were examined to assess morphological variation.

Male ants were collected primarily using Malaise traps and Lindgren funnel traps, which are effective for capturing alate males during nuptial flight periods. Additional specimens were obtained using blacklight and mercury vapor lamps, UV traps, pitfall traps, yellow pan traps, box traps, and sweep netting.

A supplementary dataset (Suppl. material 1) is provided, including detailed collection information and unique identifiers for examined specimens.

### Imaging and morphological study

Morphological observations were conducted using a Leica MZ12.5 stereomicroscope. For MEM specimens, images were captured with a Leica DFC495 digital camera mounted on a Leica Z16 microscope equipped with motorized z-stepping, and image stacks were combined using Leica Application Suite v. 4.1.0 (Montage Module). For CAS specimens, montage images were produced using either a JVC KY-F75 digital camera with Syncroscopy Auto-Montage v. 5.0 or a Leica DFC425 camera with Leica Application Suite v. 3.8.

All specimen images are available through AntWeb (antweb.org) and can be accessed using their unique specimen identifiers (MEM codes or CASENT numbers).

### Depositories

Specimens examined in this study are deposited in the following institutions:

**ABS** Archbold Biological Station Collection, Venus, Florida, USA;

**CAS** California Academy of Sciences, San Francisco, CA, USA;

**FMNH** Field Museum of Natural History, Chicago, Illinois, USA;

**JTLC** John T. Longino Collection, USA;

**LACM** Natural History Museum of Los Angeles County, Los Angeles, California, USA;

**MCZ** Museum of Comparative Zoology, Harvard University, Cambridge, Massachusetts, USA;

**MEM** Mississippi Entomological Museum, Mississippi State Univ., Starkville, Mississippi, USA;

**UCDC** Bohart Museum of Entomology, University of California, Davis California, USA;

**USNM** National Museum of Natural History, Smithsonian Institution Washington, D.C., USA;

## Results

### Synoptic list of Formicinae Latreille, 1809 genera in the Nearctic region

Genera in bold are represented in the Nearctic solely by introduced species.

*Acropyga* Roger, 1862

*Acropyga epedana* Snelling, 1973

***Anoplolepis*** Santschi, 1914

***Anoplolepis gracilipes*** (Smith, 1857)

*Brachymyrmex* Mayr, 1868

*Camponotus* Mayr, 1861

*Colobopsis* Mayr, 1861

*Formica* Linnaeus, 1758

*Lasius* Fabricius, 1804

*Myrmecocystus* Wesmael, 1838

***Myrmelachista*** Roger, 1863

***Myrmelachista ramulorum*** Wheeler, 1908

*Nylanderia* Emery, 1906

***Paratrechina*** Motschoulsky, 1863

***Paratrechina longicornis*** (Latreille, 1802)

***Plagiolepis*** Mayr, 1861

***Plagiolepis alluaudi*** Emery, 1894

*Polyergus* Latreille, 1804

*Prenolepis* Mayr, 1861

*Prenolepis imparis* (Say, 1836)

### Diagnosis of male ants of the subfamily Formicinae in the Nearctic region

- **Antenna with 10–13 segments**; scape reaching or exceeding posterior margin of head, bearing erect macrosetae or not; antennal insertion confluent or distant to posterior margin of clypeus; pedicel subequal to first flagellomere in length and diameter, or shorter and broader; flagellum shorter or longer than mesosoma.
- Posteromedian margin of clypeus not produced backwards between antennal insertion.
- Mandible reduced and short, narrow and falcate, well-developed, triangular.
- Masticatory margin of mandible never serrate, edentate to dentate (with one to five teeth and up to eight or nine denticles), triangular to falcate.
- Mesopleural oblique furrow always present, reaching pronotum far from pronotal posteroventral margin.
- Metatibia with one spur or no spur.
- Palp formula 4:2, 5:3, or 6:4.
- Petiole attached ventrally to first gastral segment; petiole much smaller than first gastral segment in lateral view.
- Fourth abdominal segment without cinctus (constriction) between pre- and postsclerites.
- Apical portion of abdominal sternum IX not bi-spinose.
- Basimere weakly developed, usually indistinct from and usually approx. the same size as telomere. Telomere extending anteroventrally beneath basimere almost to base of paramere.
- Forewing venation reduced: cross-vein 2rs-m absent, thus submarginal cell 2 open.
- Jugal lobe absent.
- Pretarsal claws simple or with small subapical tooth.
- Pygostyles present, varying in length.
- Scuto-scutellar suture simple.
- **Wings absent or fully developed**. Marginal cell 1 length less than one-third chord length of wing.

### Morphological analysis

**Figure 1.**
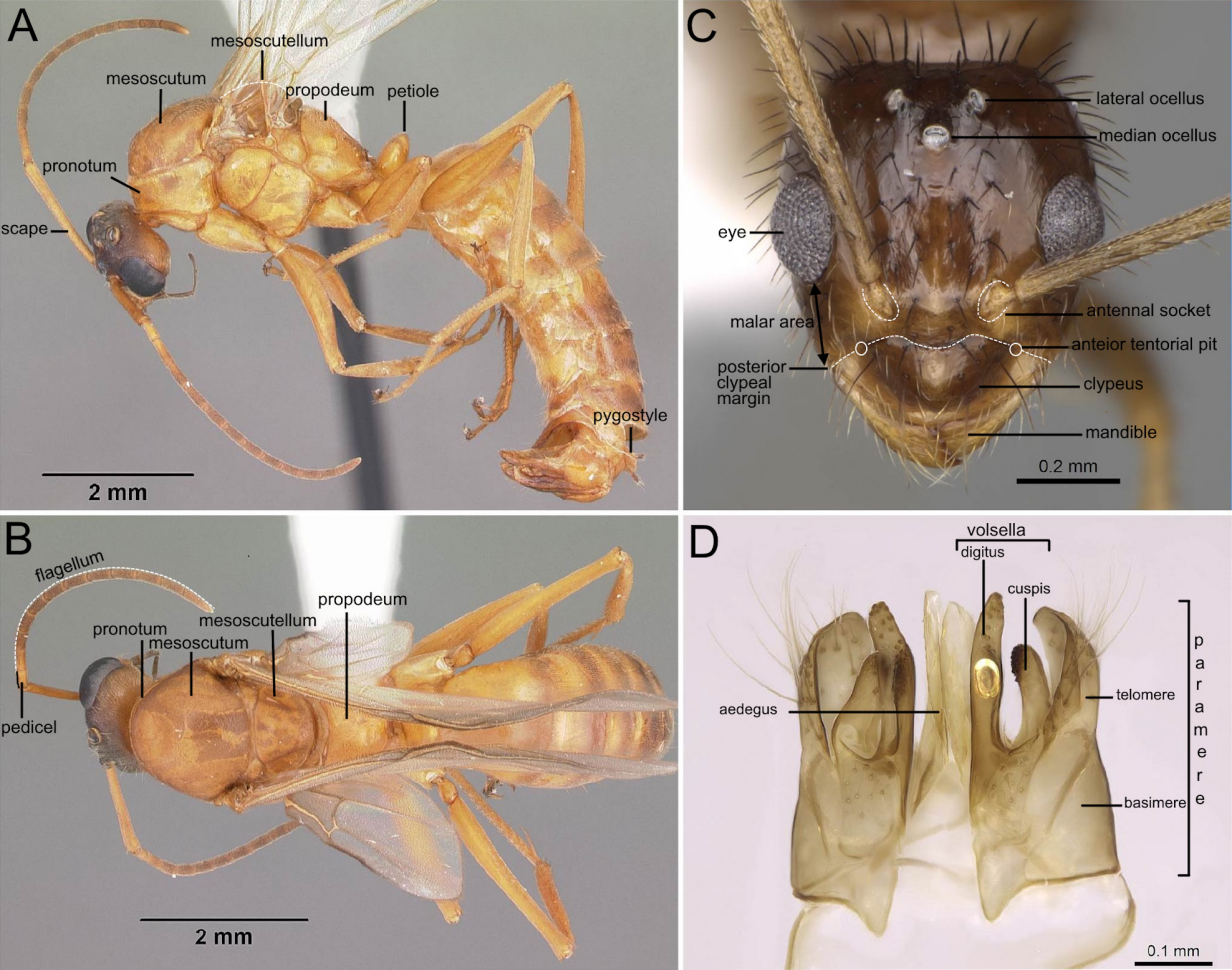
**A**. Body in lateral view. *Formica pallidefulva* (CASENT0103929; photo by April Nobile; AntWeb.org); **B**. Body in dorsal view. *Formica pallidefulva* (CASENT0103929; photo by April Nobile; AntWeb.org); **C**. Head in full-face view. *Nylanderia hystrix* (CASENT0106724; photo by Michele Esposito; AntWeb.org); **D**. Genitalia in ventral view. *Paratrechina longicornis* (CASENT0740916; photo by Veronica M. Simote; AntWeb.org).

List of characters:

1. The number of antennal segments: 10–13.
2. The length of scape (Fig. 1A): scape reaching or exceeding posterior margin of head.
3. The position of antennal insertion: close to the posterior margin of the clypeus (distance < diameter of scape) to distant from the posterior margin of the clypeus (distance ≥ diameter of scape).
4. The length of funiculus: shorter to longer than mesosoma length.
5. The shape of mandible: reduced or well-developed: falcate and edentate to broadly triangular and dentate.
6. The number of teeth on masticatory margin: 0–9.
7. The length of malar space (Fig. 1C): extremely reduced (shorter than median clypeal length) to well developed (as long or longer than median clypeal length).
8. The form of anterior clypeal margin: straight to weakly concave or with a convex lobe.
9. The form of posterior clypeal margin (Fig. 1C): slightly convex or oblique laterally, with a straight to weakly concave median section.
10. The position of lateral ocelli either breaking the occipital margin or entirely within the vertex.
11. The length of maxillary palp: shorter than the head or approx. as long as the head.
12. Psammophore: present or absent.
13. The position of anterior tentorial pit: adjacent to or distinctly set apart from the antennal sockets.
14. Wings: present and fully developed or highly reduced or absent.
15. On the forewings (Fig. 2A): marginal cell open or closed.
16. On the hindwing (Fig. 2B): Media (M), M+Cu, and free section of cubitus present.
17. Macrosetae on mesonotum dorsum: absent or present.
18. Propodeum (Fig. 1A, B): propodeum with dorsal face shorter than declivitous face; or dorsal and declivitous faces subequal, meeting at an obtuse angle.
19. Propodeal spiracle: small and circular or variable in size (from small and circular to large and slit-shaped).
20. Petiole: low and flattened to high node with distinct front (Fig. 1A), dorsal, and rear faces; narrowly attached to abdominal segment III.
21. Color of gastral segment varies.
22. Pygostyle (Fig. 1A): short or very long.
23. Pubescence dense or dilute.
24. Parameres (Fig. 1D): long and rectangular or short and circular; broadly triangular to well developed or short and robust; narrowly triangular or roughly triangular; densely covered in macrosetae; slight mesad curvature at the posterior end.
25. Digiti (Fig. 1D): narrow and tubular.
26. Cuspi (Fig. 1D): broad anteriorly and narrow laterally at the posterior end; small and tubular, reaching digiti dorsally; fine, forming a transverse lobe (rarely digitate in ventral view); robust, usually digitiform in ventral view.
27. Volsellar lobes (Fig. 1D): flat and slightly indented relative to the digital margin; digitus scoop-shaped in anteroventral view, with the apex appearing “folded” laterally.
28. Pretarsal claws: simple or with a small subapical tooth.

### Wing venation

**Figure 2.**
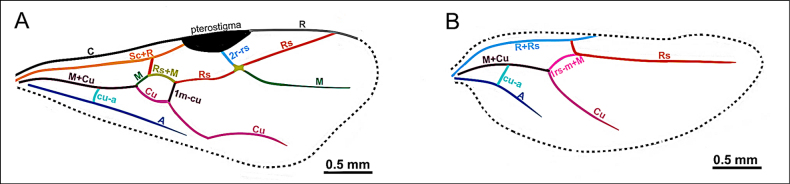
Illustration of wing venation in Formicinae males. **A**. Forewing of *Polyergus breviceps* (CASENT0100665); **B**. Hindwing of *Polyergus vinosus* (CASENT0281081). Abbreviations: C = Costa, Sc+R = Subcostal+Radial, Rs = Radial Sectorial, 2r-rs = second radial-radiosectorial, M+Cu = Media+Cubital, M = Media, Rs+M = Radial sectorial+Media, 1m-cu = first media-cubital, Cu = Cubital, cu-a = cubital-anal, A = Anal, 1rs-m+M = radial sectorial-media + Media.

### Key to genera of the subfamily Formicinae based on males

**Table:** 

1	Antenna with 10 to 11 segments (Fig. 3A)	**2 (Tribe Myrmelachistini)**
–	Antenna with 12 to 13 segments (Fig. 3B)	**3**
2(1)	Antenna with 10 segments; apical segments gradually thickened toward apex (Fig. 4A). Mesoscutellum anteriorly extended, projecting over posterior margin of pronotum (Fig. 4B). Propodeum with dorsal face distinctly shorter than declivitous face (Fig. 4B). Petiole low, flattened (Fig. 4B). Forewing marginal cell open (Fig. 4C). Mandible reduced; lateral ocelli breaking occipital margin (Fig. 4A)	*** Brachymyrmex ***
–	Antenna with 10 or 11 segments; when 10 segments, apical 4 segments clavate (Fig. 4D). Mesoscutellum not anteriorly extended over posterior margin of pronotum (Fig. 4E). Propodeum with dorsal and declivitous faces subequal, meeting at obtuse angle (Fig. 4E). Petiole nodiform, with distinct anterior, dorsal, and posterior faces (Fig. 4E). Forewing marginal cell closed (Fig. 4F). Mandible well developed; lateral ocelli not breaking occipital margin (Fig. 4D)	*** Myrmelachista ***
3(1)	Antenna with 12 segments (Fig. 5A)	**4**
–	Antenna with 13 segments (Fig. 5B)	**7**
4 (3)	Masticatory margin of mandible with 8–9 teeth (Fig. 6A). Scape long, surpassing posterior margin of head by more than one-third its length (Fig. 6B). Pterostigma narrow, elongate (Fig. 6C)	***Anoplolepis gracilipes***
–	Masticatory margin of mandible with fewer than 5 teeth (Fig. 6D). Scape short, not reaching or surpassing posterior margin of head by at most one-third its length (Fig. 6E). Pterostigma broad, short (Fig. 6F)	**5**
5 (4)	Body with dense, appressed pubescence. Petiole higher than long, nodiform (Fig. 7A). Masticatory margin with 2 teeth (Fig. 7A). Parameres elongate, apically falciform (Fig. 7B)	***Acropyga epedana***
–	Body without dense appressed pubescence; pubescence sparse to absent. Petiole low, squamiform (Fig. 7C). Parameres short, apically digitiform and rounded (Fig. 7D)	**6**
6(5)	Malar space extremely reduced, shorter than median clypeal length (Fig. 8A). Masticatory margin of mandible with 2 or 3 teeth. Scape slightly surpassing posterior margin of head by approx. length of pedicel. Flagellum shorter than mesosoma. Dorsum of mesosoma lacking macrosetae (Fig. 8B)	***Plagiolepis alluaudi***
–	Malar space distinctly longer than median clypeal length (Fig. 8C). Masticatory margin of mandible with single apical tooth. Scape long, surpassing posterior margin of head by length of pedicel plus first 2 flagellomeres. Flagellum longer than mesosoma. Dorsum of mesosoma with abundant macrosetae (Fig. 8D)	***Nylanderia parasitica***
7(3)	Frons with macrosetae present (Fig. 9A)	**8 (Tribe Lasiini)**
–	Frons without macrosetae (Fig. 9B)	**12**
8 (7)	Scape short, not exceeding or only slightly exceeding posterior margin of head. Macrosetae present, scattered on frons and head dorsum (Fig. 10A). Parameres elongate, slender (Fig. 10B)	***Prenolepis imparis***
–	Scape long, distinctly surpassing posterior margin of head. Macrosetae abundant on head dorsum (Fig. 10C). Parameres short, robust to broadly triangular (Fig. 10D)	**9**
9(8)	Wings absent or highly reduced (Fig. 11A)	**10**
–	Wings present and fully developed (Fig. 11B)	**11**
10(9)	Gaster light brown in color, similar to mesosoma; REL 27–28, SI 112–121 (Fig. 12A)	***Nylanderia deyrupi***
–	Head and gaster dark brown in color contrasting with light brown mesosoma; REL 34–36, SI 125–127 (Fig. 12B)	***Nylanderia deceptrix***
11(9)	Scape long, surpassing posterior margin of head by more than one-half its length. Maxillary palp approx. as long as head (Fig. 13A). Parameres short, robust (Fig. 13B)	***Paratrechina longicornis***
–	Scape shorter, surpassing posterior margin of head by less than one-half its length. Maxillary palp shorter than head (Fig. 13C). Parameres broadly triangular to digitate (Fig. 13D)	***Nylanderia* (part)**
12 (7)	Posterior clypeal margin slightly convex. Anterior tentorial pits adjacent to antennal sockets (Fig. 14A)	**13**
–	Posterior clypeal margin oblique laterally, median section straight to weakly concave between antennal sockets. Anterior tentorial pits distinctly separated from antennal sockets, near midlength of clypeus (Fig. 14B)	**14**
13 (12)	Maxillary palp greatly elongate. Psammophore present. Scape very long, surpassing posterior margin of head by more than twice length of pedicel (Fig. 15A)	*** Myrmecocystus ***
–	Maxillary palp short. Psammophore absent. Scape short, not exceeding or only slightly exceeding posterior margin of head (Fig. 15B)	*** Lasius ***
14(12)	With the head in full-face view, antennal insertion close to posterior margin of clypeus; with the distance between them less than the diameter of the scape (Fig. 16A)	**15 (Tribe Formicini)**
–	With the head in full-face view, antennal insertion distant to posterior margin of clypeus, with the distance between them approx. equal to or greater than the diameter of the scape (Fig. 16B)	**16 (Tribe Camponotini)**
15(14)	Mandibles falcate, edentate; anterior clypeal margin straight to weakly concave; scape short, just reaching posterior margin of head (Fig. 17A); pygostyles short in posterior view of gaster (Fig. 17B)	*** Polyergus ***
–	Mandibles broadly triangular, dentate; anterior clypeal margin with convex lobe; scape long, surpassing posterior margin of head (Fig. 17C); pygostyles very long in posterior view of gaster (Fig. 17D)	*** Formica ***
16(14)	Distance between anterior tentorial pits greater than distance between frontal carinae. Frontal carinae distinctly margined, weakly diverging posteriorly (Fig. 18A). Petiolar node variable, nodiform to squamiform; posterodorsal margin with standing setae (Fig. 18B)	*** Camponotus ***
–	Distance between anterior tentorial pits approximately equal to distance between frontal carinae. Frontal carinae irregular, strongly diverging posteriorly (Fig. 18C). Petiolar node nodiform; posterodorsal margin without standing setae (Fig. 18D)	*** Colobopsis ***

**Figure 3.**
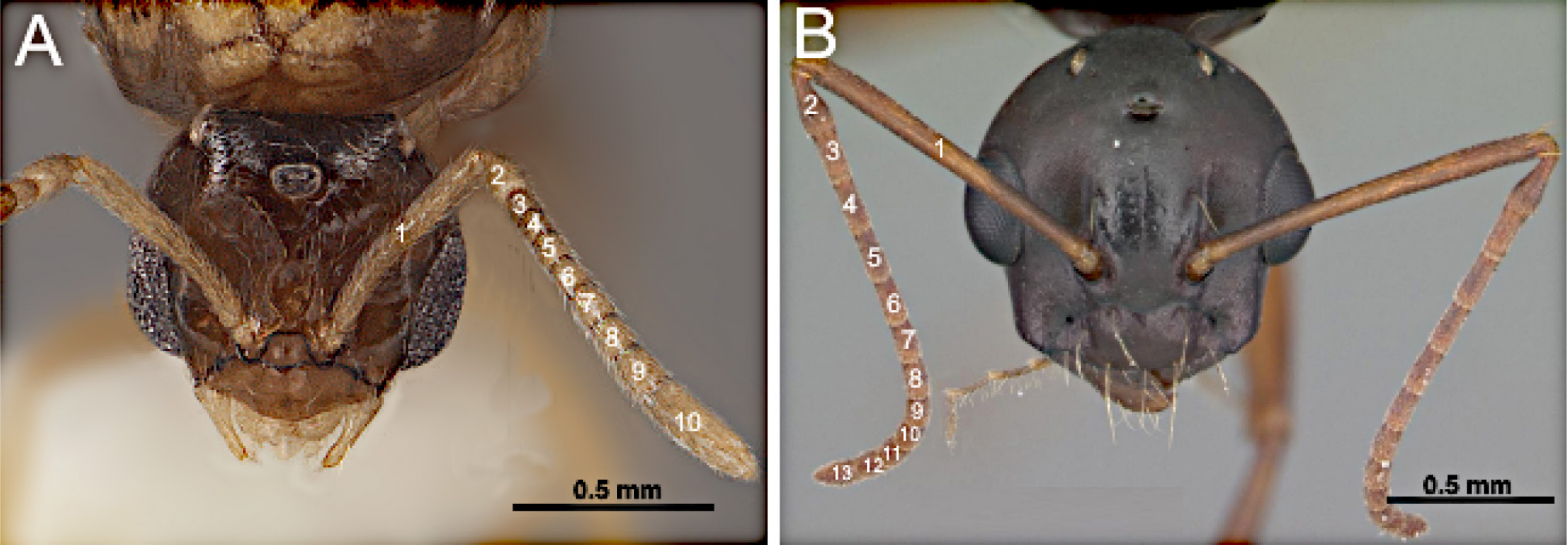
Head in full-face view. **A**. *Brachymyrmex patagonicus* (MEM 214314; photo by Joe MacGown); **B**. *Camponotus decipiens* (CASENT0103662; photo by April Nobile; AntWeb.org).

**Figure 4.**
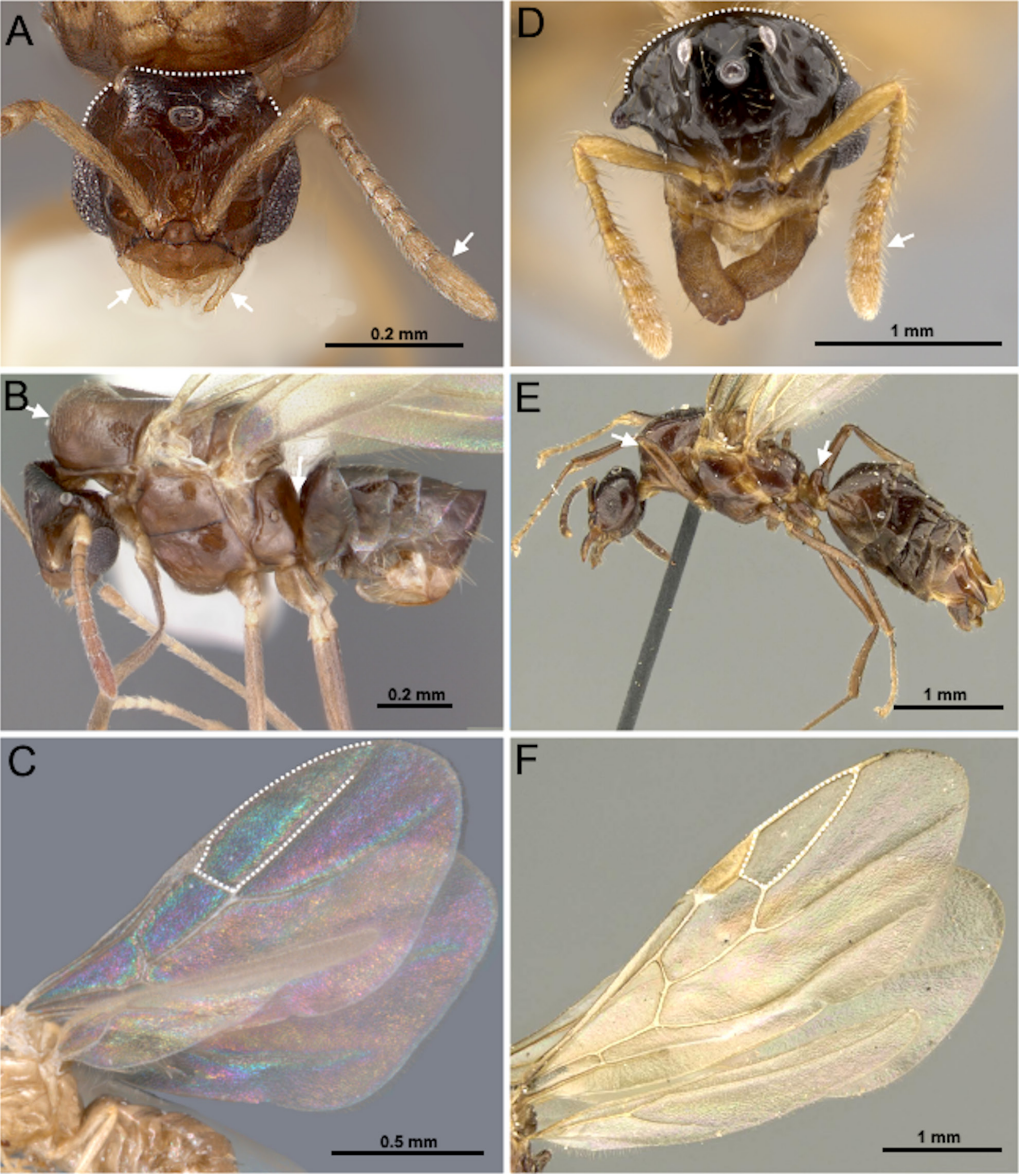
Body in lateral view and head in full-face view. **A–C**. *Brachymyrmex patagonicus* (MEM 214314; photo by Joe MacGown); **D**. *Myrmelachista* rsp069 (CASENT0654158; photo by J. Longino; AntWeb.org); **E–F**. *Myrmelachista catharinae* (CASENT0915738; photos by Daniela Lehner; AntWeb.org).

**Figure 5.**
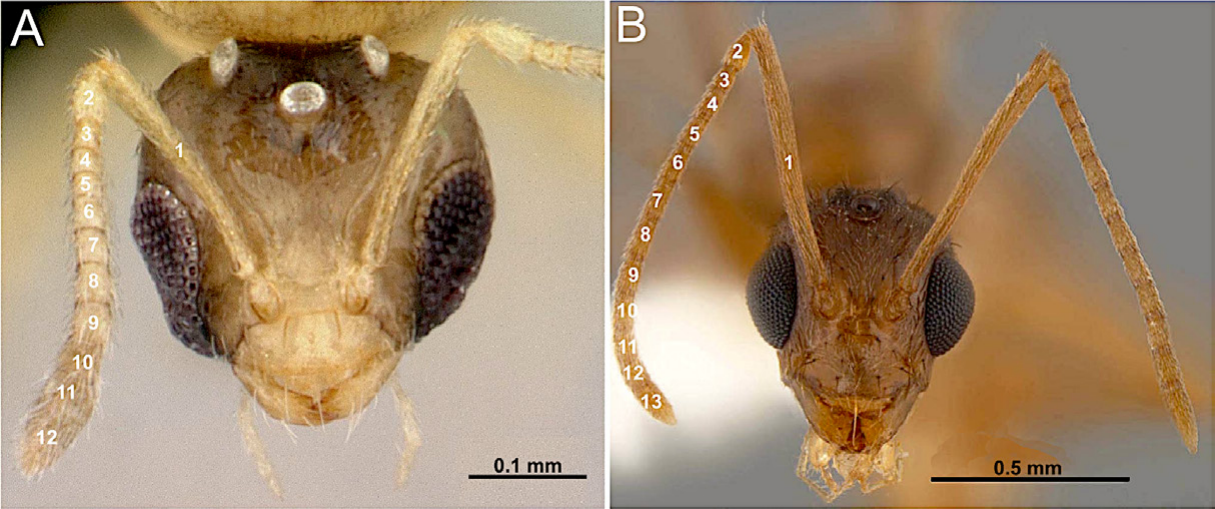
Head in full-face view. **A**. *Plagiolepis alluaudi* (CASENT0495472; photo by Erin Prado; AntWeb.org); **B**. *Nylanderia fulva* (MEM 213583; photo by Joe MacGown).

**Figure 6.**
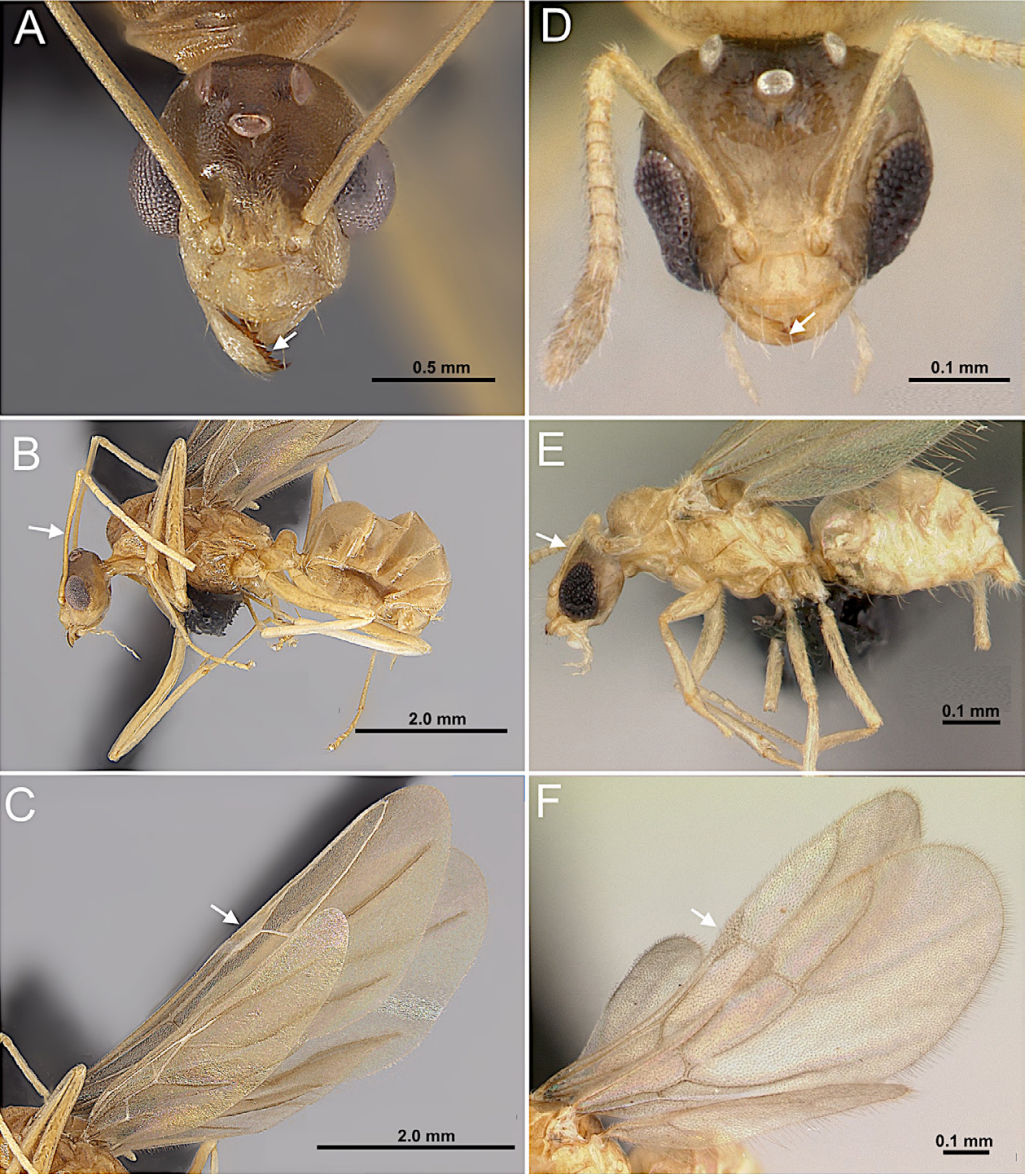
Head in full-face view, body, and forewing in lateral view. **A–C**. *Anoplolepis gracilipes* (MEM 206500; photo by Joe MacGown); **D, E**. *Plagiolepis alluaudi* (CASENT0495472; photos by Erin Prado; AntWeb.org).

**Figure 7.**
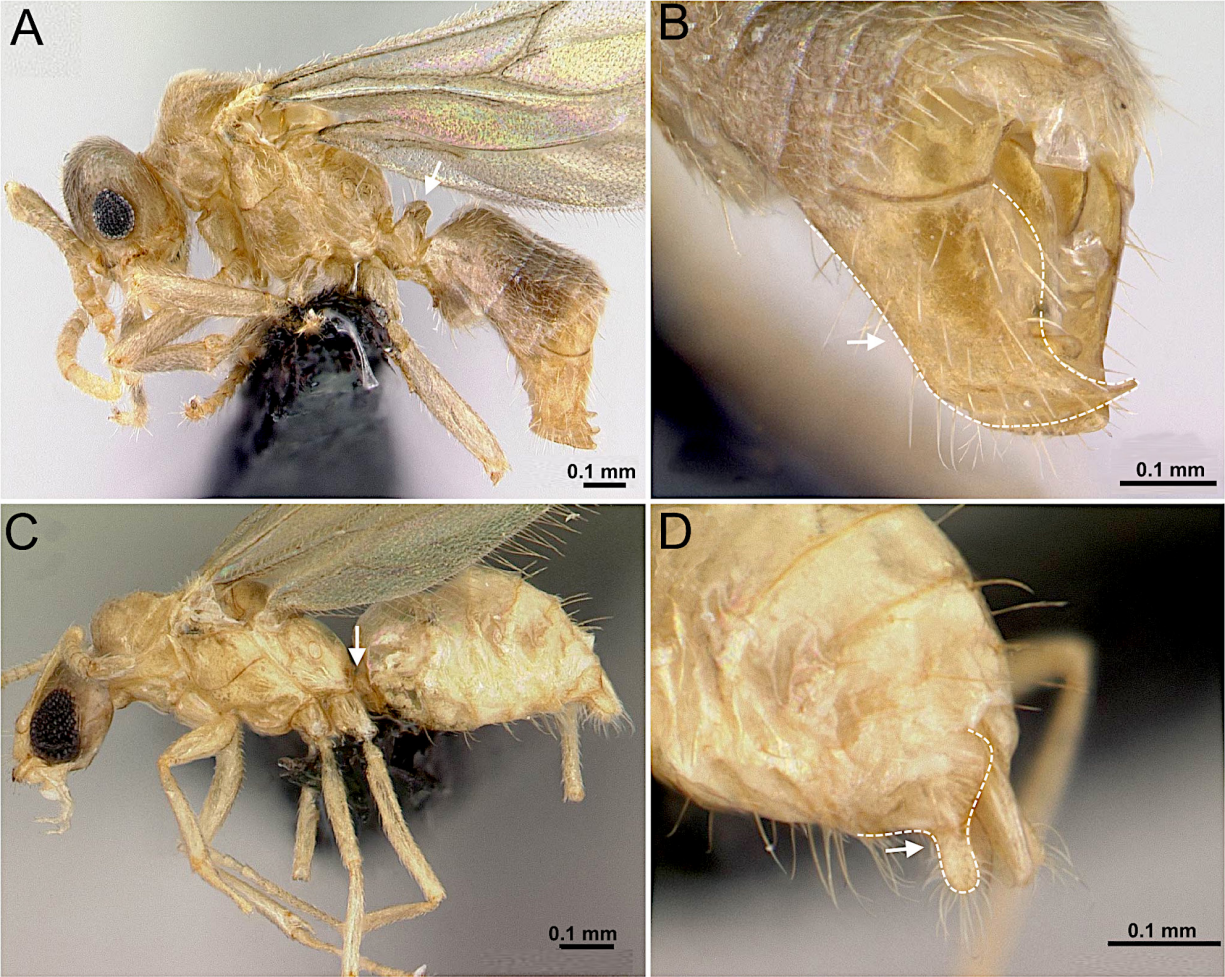
Body in lateral view and pygidium in rear view. **A, B**. *Acropyga epedana* (CASENT0188857; photos by Erin Prado; AntWeb.org); **C, D**. *Plagiolepis alluaudi* (CASENT0495472; photos by Erin Prado; AntWeb.org).

**Figure 8.**
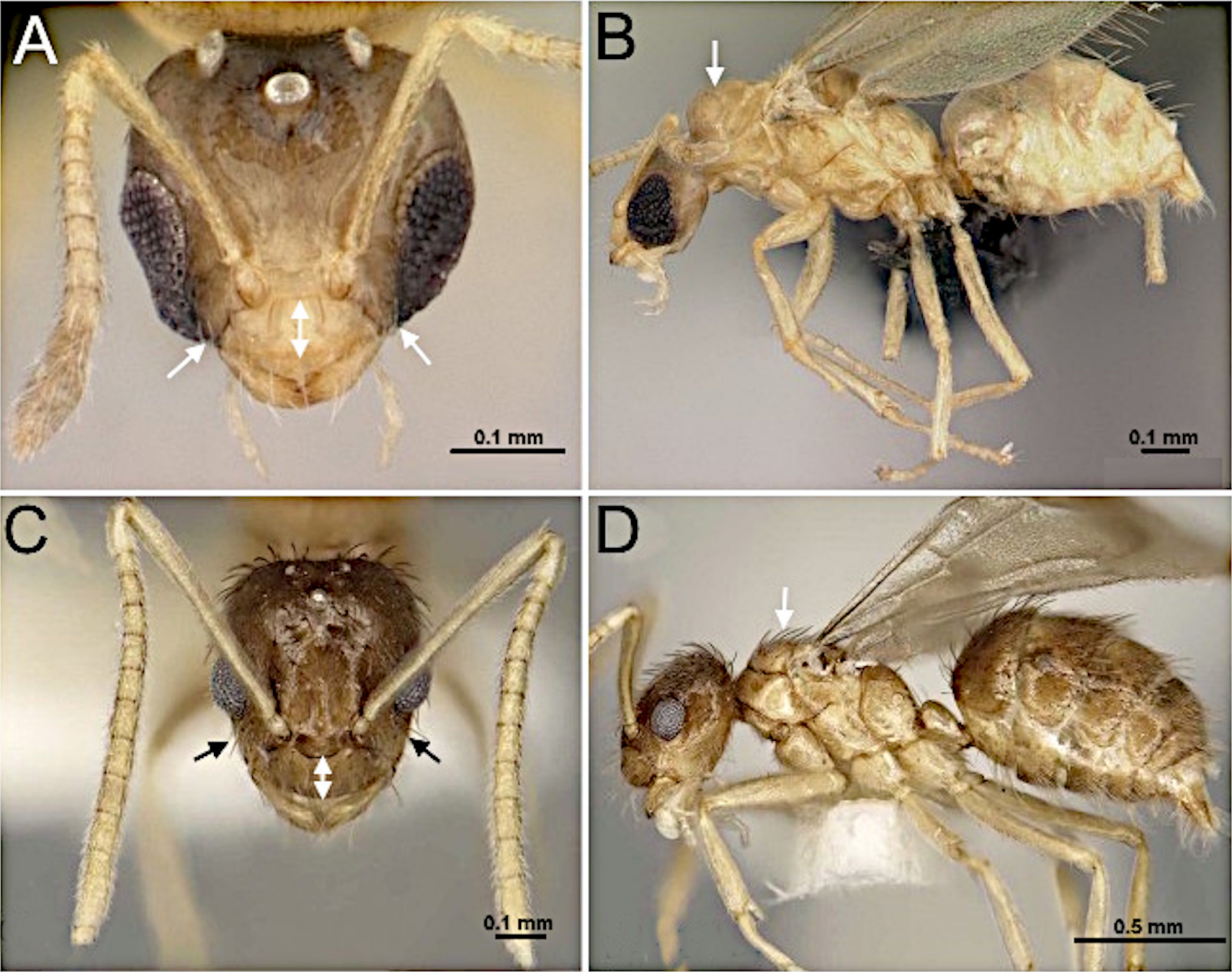
Head in full-face view and body in lateral view. **A, B**. *Plagiolepis alluaudi* (CASENT0495472; photo by Erin Prado; AntWeb.org); **C, D**. *Nylanderia parasitica* (images from Messer et al. 2020).

**Figure 9.**
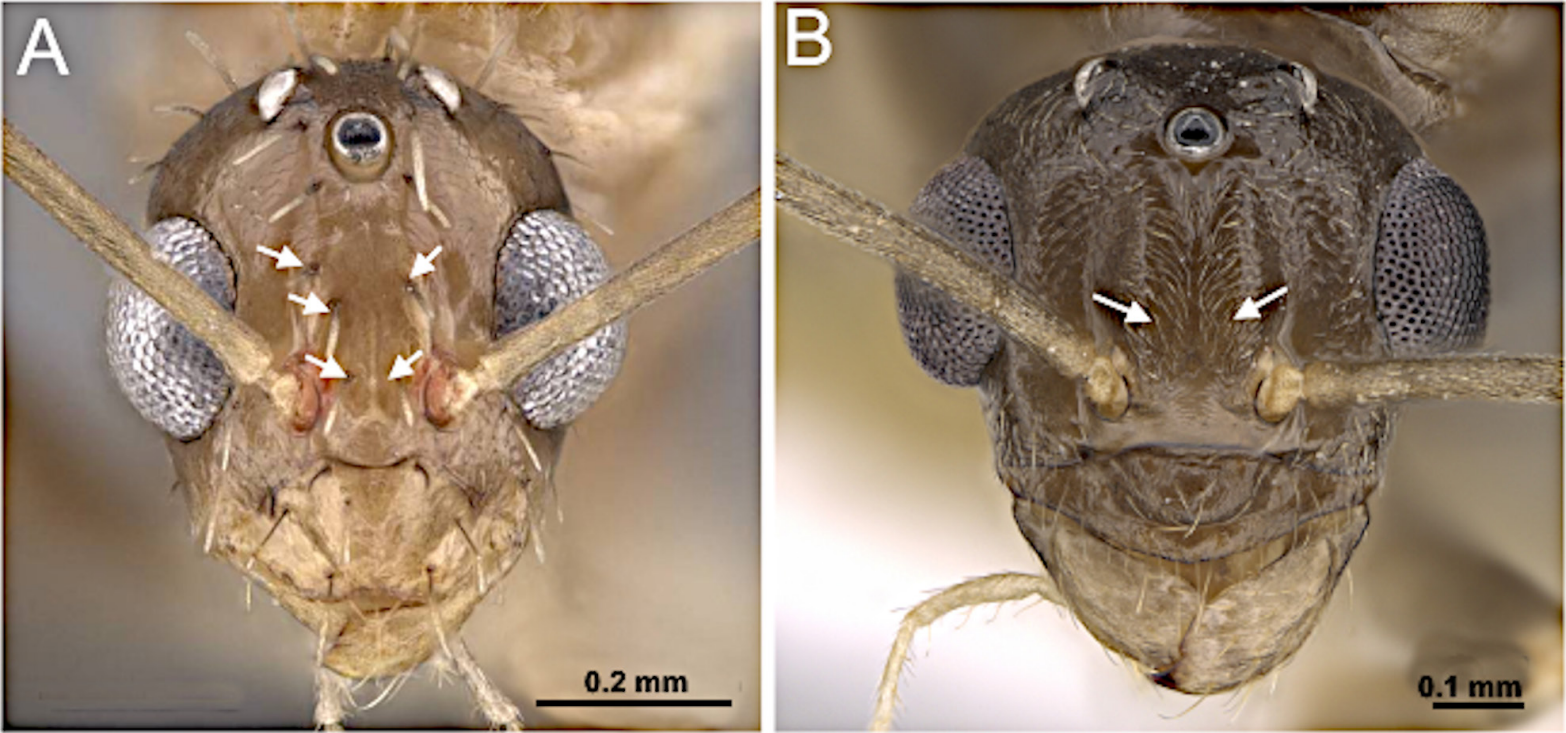
Head in full-face view. **A**. *Paratrechina longicornis* (CASENT0740916; photo by Veronica M. Sinotte; AntWeb.org); **B**. *Myrmecocystus mexicanus* (CASENT0862010; photo by Wade Lee; AntWeb.org).

**Figure 10.**
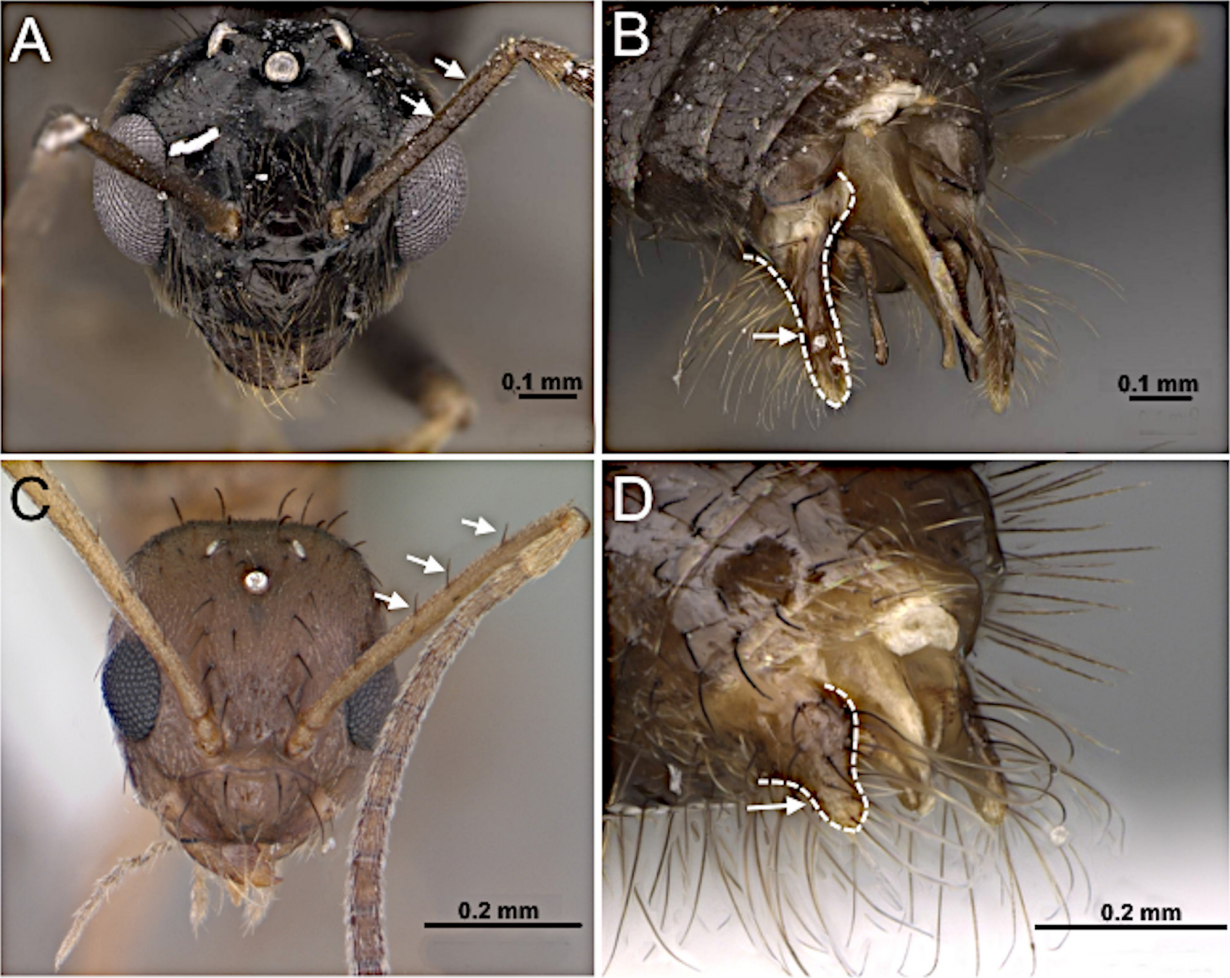
Head in full-face view and posterior portion of abdomen in posterolateral view. **A, B**. *Prenolepis imparis* (CASENT0770620; photos by Wade Lee; AntWeb.org); **C**. *Nylanderia concinna* (CASENT0104211; photo by April Nobile; AntWeb.org); **D**. *Nylanderia hystrix* (CASENT0106724; photo by Michele Esposito; AntWeb.org).

**Figure 11.**
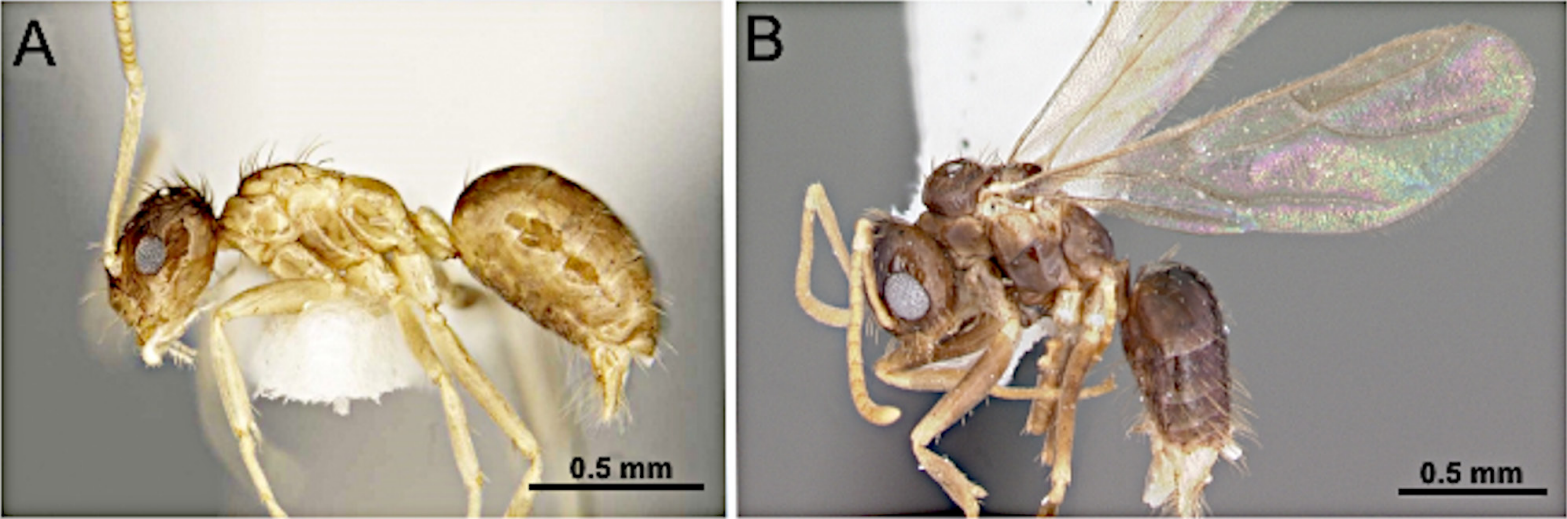
Body in lateral view. **A**. *Nylanderia deyrupi* (Image reproduced from Messer et al. (2020), with permission); **B**. *Nylanderia phantasma* (CASENT0104243; photo by April Nobile; AntWeb.org).

**Figure 12.**
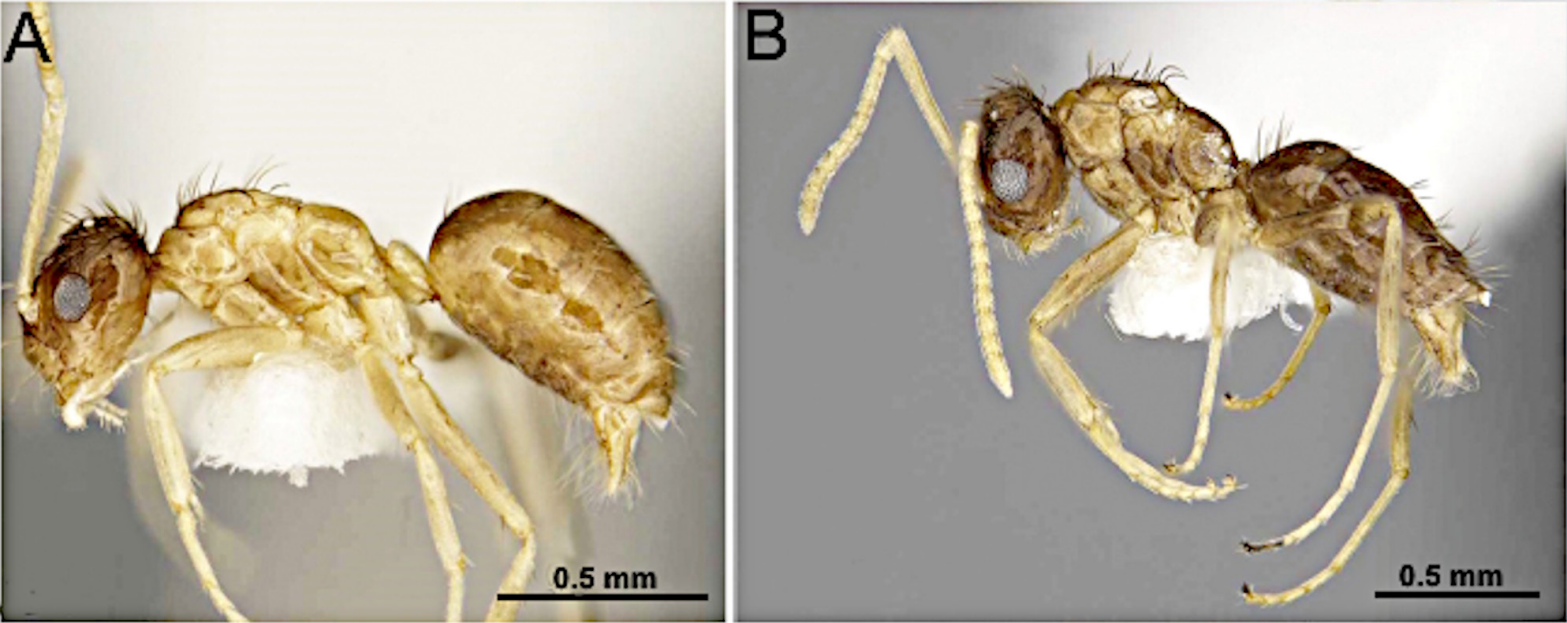
Body in lateral view. **A**. *Nylanderia deyrupi*; **B**. *Nylanderia deceptrix*. (Images reproduced from Messer et al. (2020), with permission).

**Figure 13.**
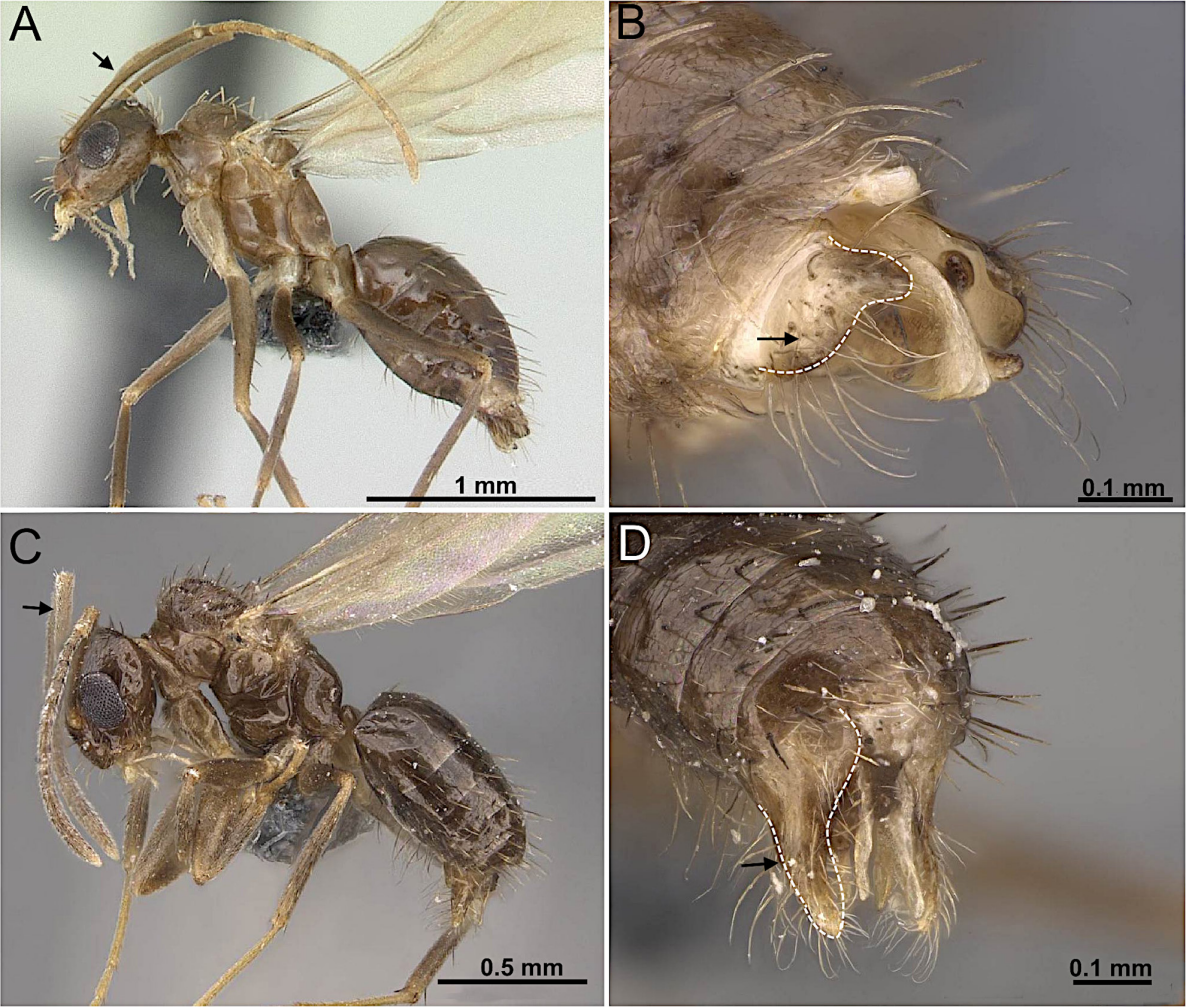
Body in lateral view and pygidium in rear view. **A, B**. *Paratrechina longicornis* (**A**. CASENT0137341; photo by Erin Prado; AntWeb.org; **B**. CASENT0740917; photo by Veronica M. Sinotte; AntWeb.org); **C, D**. *Nylanderia vividula* (CASENT0770748; photos by Wade Lee; AntWeb.org).

**Figure 14.**
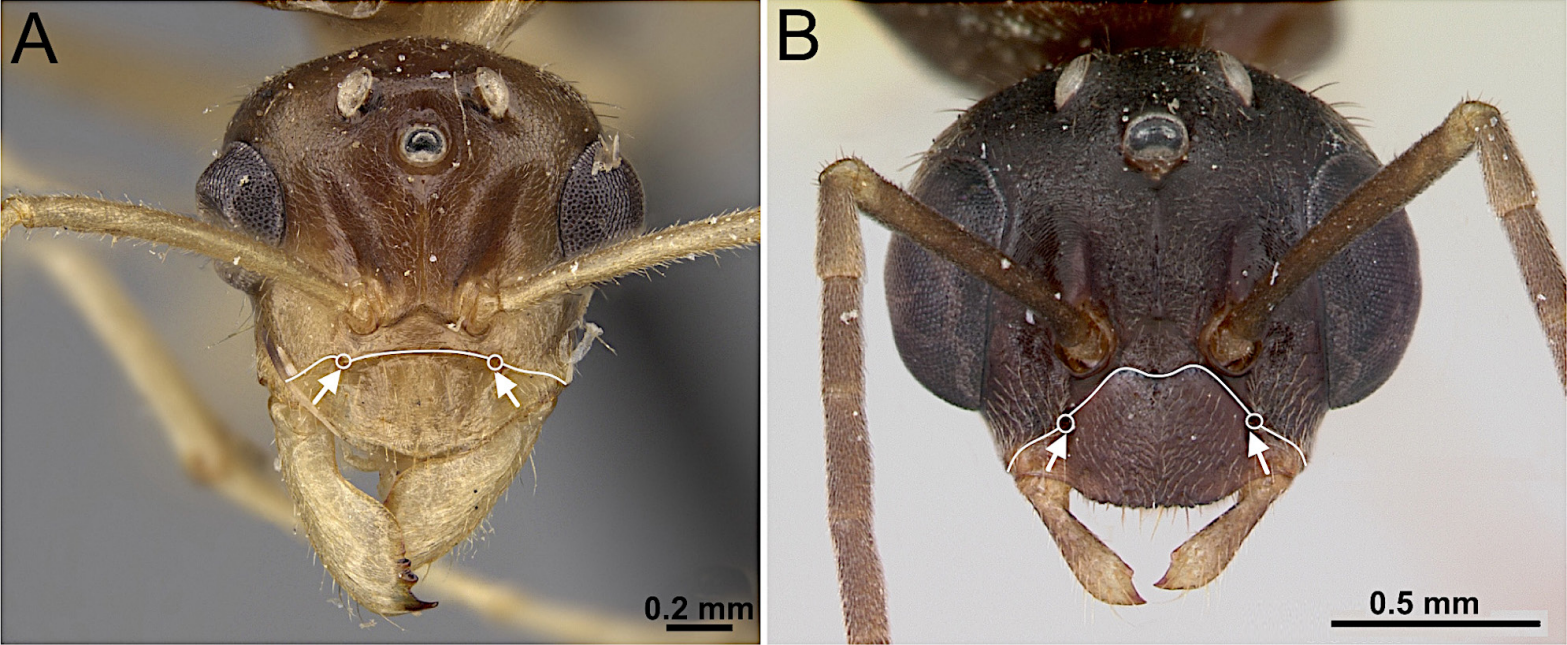
Head in full-face view. **A**. *Myrmecocystus mexicanus* (CASENT0862010; photo by Wade Lee; AntWeb.org); **B**. *Formica incerta* (CASENT0172882; photo by April Nobile; AntWeb.org).

**Figure 15.**
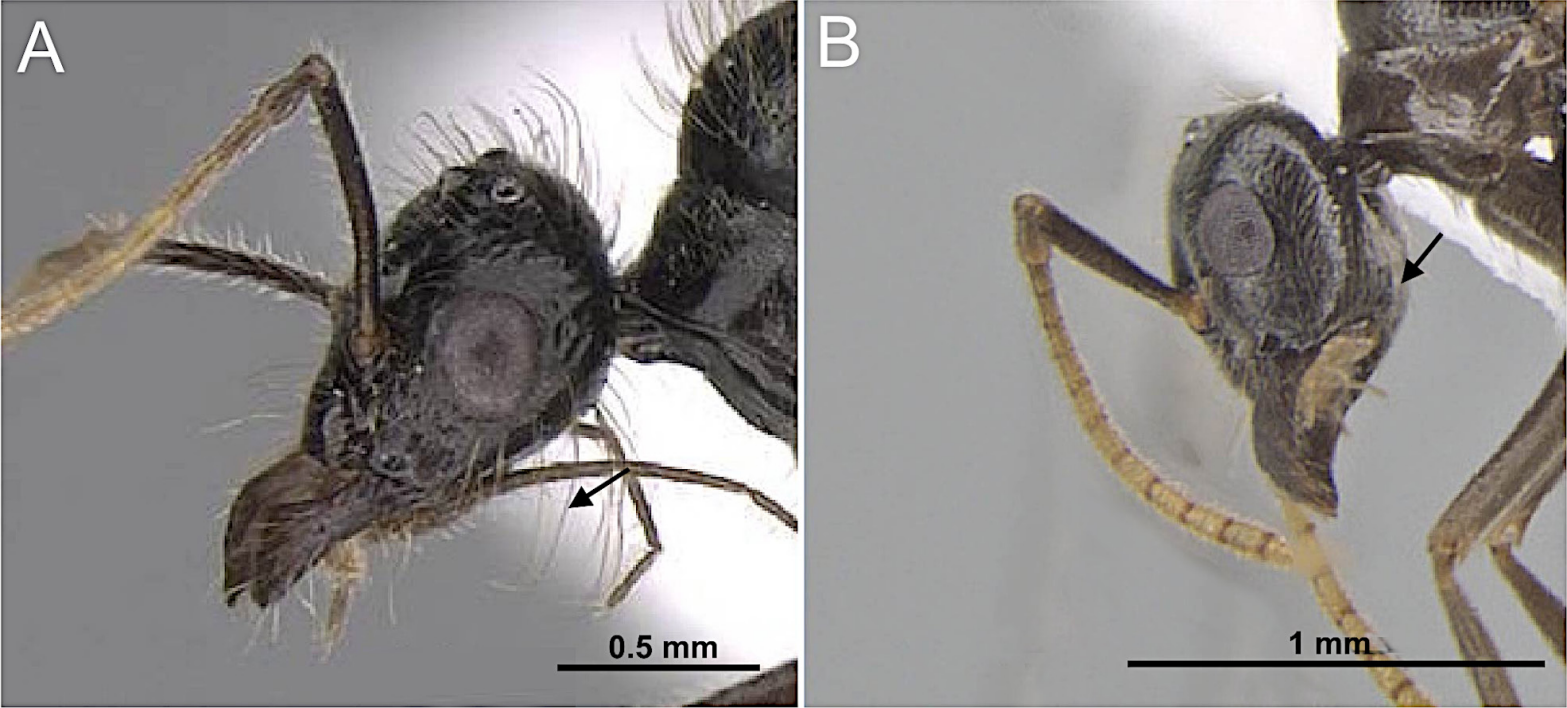
Head in lateral view. **A**. *Myrmecocystus kathjuli* (CASENT0862007; photo by Michele Esposito; AntWeb.org); **B**. *Lasius coloradensis* (CASENT0654013; photo by J. Longino; AntWeb.org).

**Figure 16.**
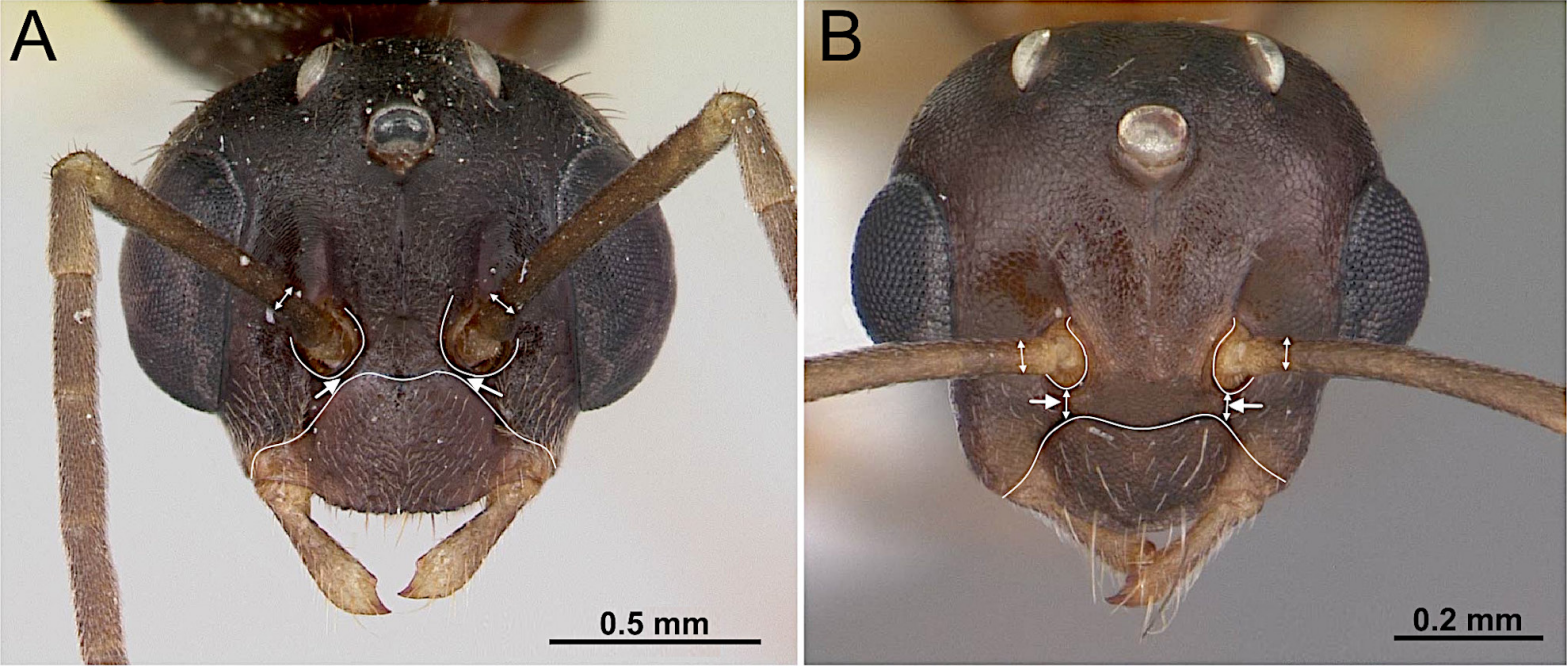
Head in full-face view. **A**. *Formica incerta* (CASENT0172882; photos by April Nobile; AntWeb.org); **B**. *Colobopsis impressa* (CASENT0103681; photos by April Nobile; AntWeb.org).

**Figure 17.**
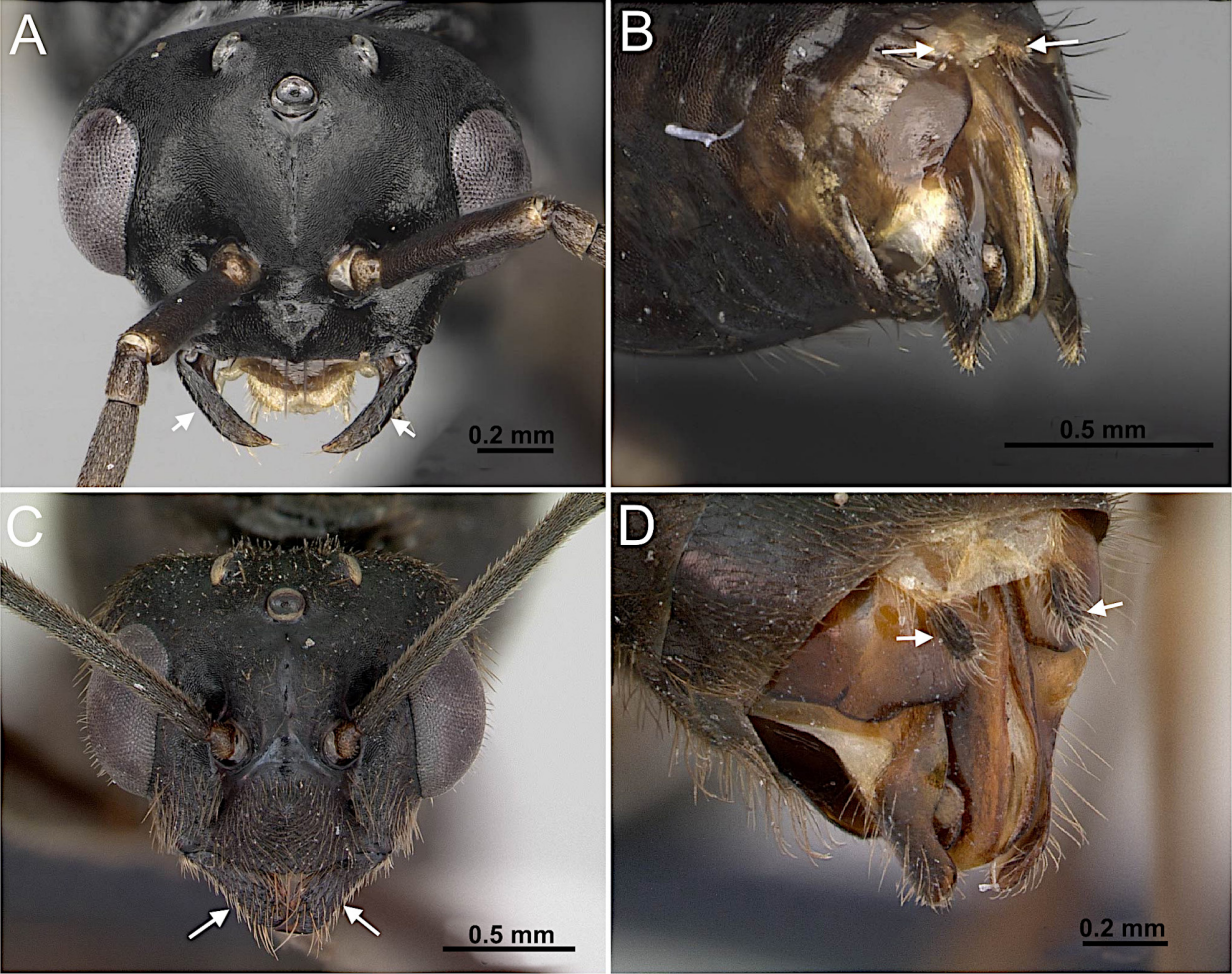
Head in full-face view and pygidium in rear view. **A, B**. *Polyergus mexicanus* (CASENT0281072; photos by Shannon Hartman; AntWeb.org); **C, D**. *Formica exsecta* (CASENT0173163; photos by April Nobile; AntWeb.org).

**Figure 18.**
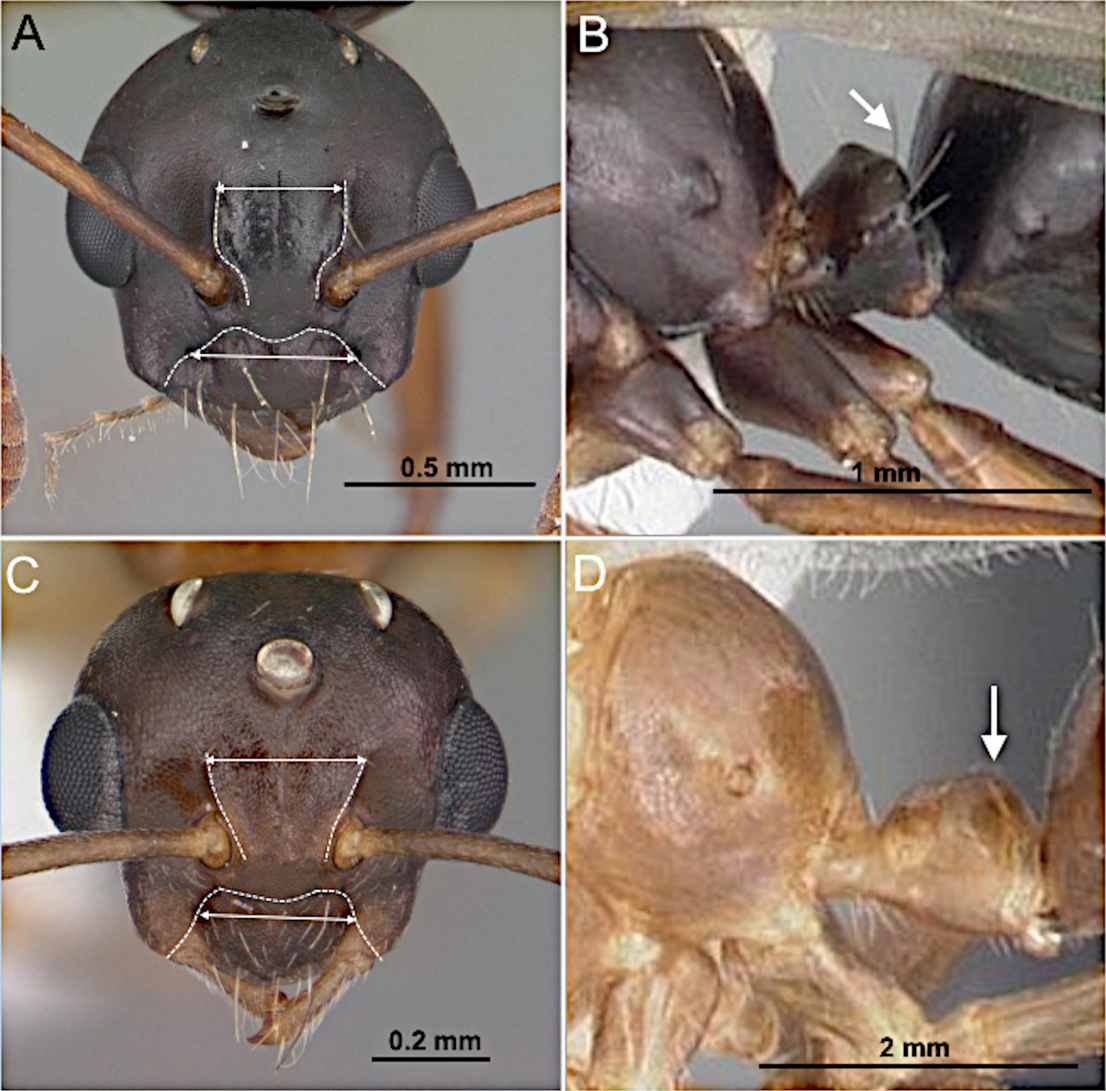
Head in full-face view and petiolar node in lateral view. **A, B**. *Camponotus decipiens* (CASENT0103662; photos by April Nobile; AntWeb.org); **C, D**. *Colobopsis impressa* (CASENT0103681; photos by April Nobile; AntWeb.org).

### Tribe Camponotini

#### Camponotus

Taxon classification

Animalia

HymenopteraFormicidae

Mayr, 1861

752B7802-9E25-578E-A096-29FFAD04CF21

##### Note.

In the Nearctic region, the genus *Camponotus* Mayr, 1861 comprises ~ 69 valid species (Bolton 2025).

##### Diagnosis.

***Head*** (Fig. 20A). Antenna with 13 segments. Medium to large-sized, overall length 5–12 mm. Mandible well developed, lobate; masticatory margin edentate or with a single apical tooth. Palp formula 6:4; maxillary palp exceeding hypostomal margin, exceeding occipital foramen or not. Antennal sockets positioned far from the posterior clypeal margin. Scape with or without erect macrosetae, subequal to or longer than head length. Funiculus shorter than mesosomal length; first flagellomere longer or shorter than second flagellomere in medial view. Frons lacking paired coarse macrosetae. Malar space with lateral margin well developed, much broader than maximum scape width.

**Figure 19.**
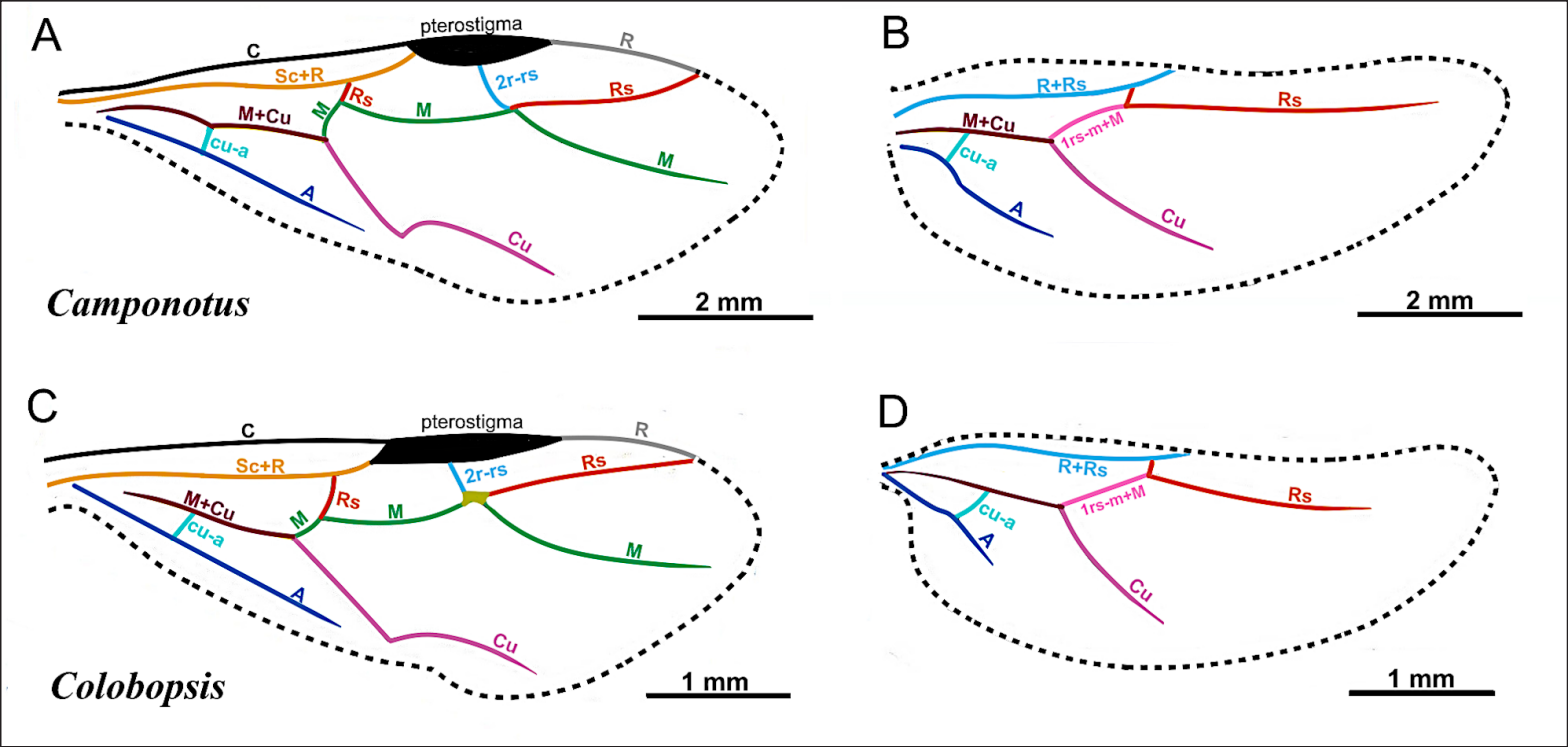
Illustration of fore- and hindwing venation in the tribe Camponotini, represented by *Camponotus* and *Colobopsis*, adapted from Antweb images (*Camponotus chromaoides*ANTWEB1060119, *Colobopsis impressa*CASENT0103681).

**Figure 20.**
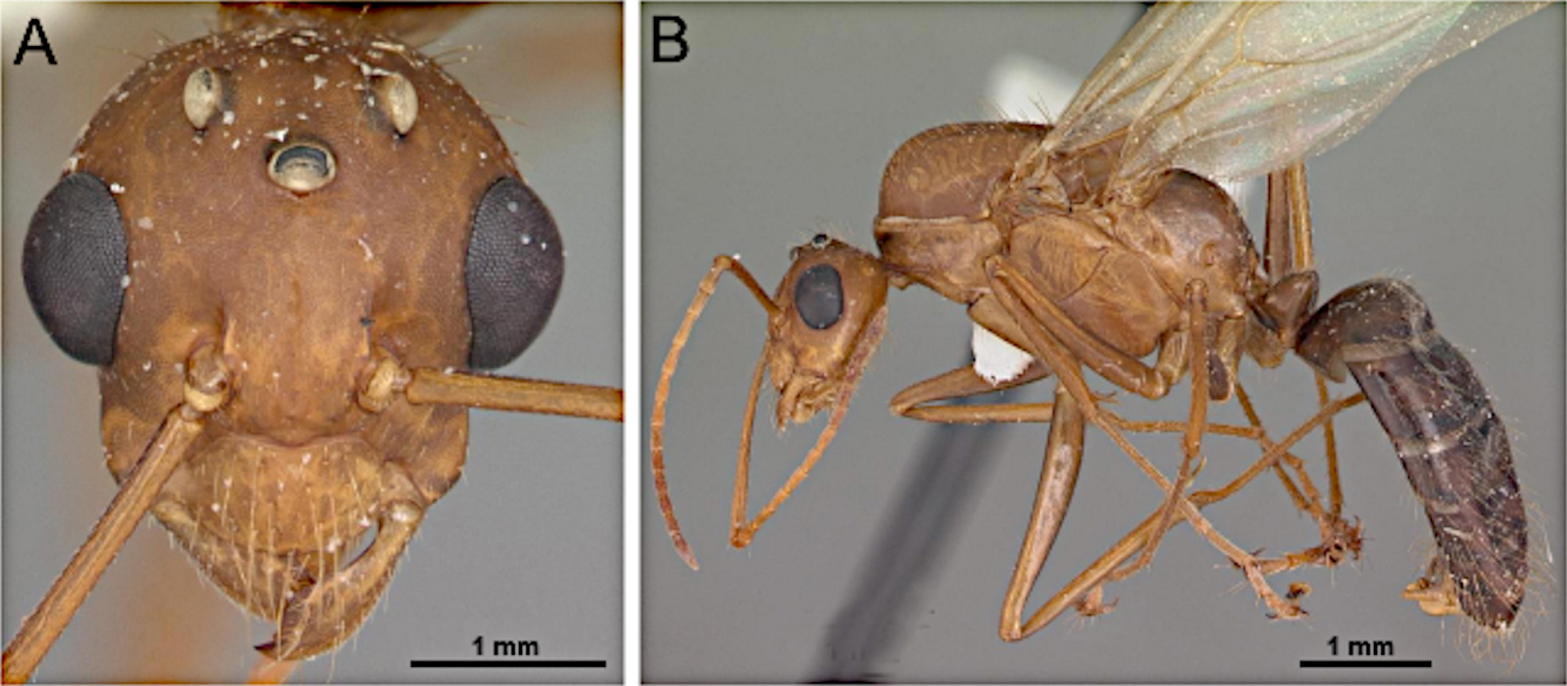
Head in full-face view (**A**) and body in lateral view (**B**) of *Camponotus floridanus* (CASENT0103676; photos by April Nobile; AntWeb.org).

***Mesosoma*** (Fig. 20B). Pronotum distinct, vertical; anterior margin of mesoscutum vertical, rounding into a flattened dorsum; mesoscutellum smoothly convex; propodeum distinct, forming a rounded angle with the declivitous face.

***Male genitalia***. Parameres elongate, apically narrowed or falciform; volsella well developed; aedeagus elongate.

***Wings*** present. ***Forewing*** (Fig. 19A): pterostigma well developed; costal vein (C) present; fused subcostal and radial veins (Sc+R) present; cross vein (2r-rs) connected with radial sector vein at the midpoint of pterostigma; radial sector-medial vein (Rs+M) absent; radial sector vein (Rs) reaching the costal margin; medial-cubital (M+Cu) vein present; first medial-cubital crossvein (1m-cu) absent; crossvein (2rs-m) absent; media vein (M) incomplete, not reaching the wing margin; cubital-anal crossvein (cu-a) proximal to junction between media and cubitus; anal vein (A) longer than the fused medial-cubital vein M+Cu. ***Hindwing*** (Fig. 19B): fused radius and radial sector vein (R+Rs) present; medial-cubital (M+Cu) vein present; fused radial sector-medial crossvein and medial vein (1rs-m+M) present; cubital-anal crossvein (cu-a) present; free section of cubitus (Cu) present; anal vein (A) longer than M+Cu.

##### Remarks.

Males of *Camponotus* are among the largest Nearctic formicines and are recognized by their robust body form, scape elongate, and well-developed wing venation. They most closely resemble *Colobopsis*, sharing antennal sockets positioned well posterior to the anterior clypeal margin, but differ in having longer scape and mesosoma. Confusion with *Formica* is possible, but *Camponotus* males possess 13 antennal segments. Genitalic structures are relatively large and provide additional diagnostic characters.

##### Biology and distribution.

*Camponotus* is widely distributed across the United States and occupies a broad range of habitats, including forests, woodlands, grasslands, deserts, and montane environments (AntWeb 2025). Species richness is highest in the eastern United States and along the Pacific Coast, with additional diversity in the Southeast, Midwest, Southwest, and western montane regions. Most species nest in soil, rotting wood, or plant cavities, and many are commonly encountered in forested habitats. Colonies are typically large, and workers forage primarily for arthropods and carbohydrate resources such as honeydew and plant exudates.

#### Colobopsis

Taxon classification

Animalia

HymenopteraFormicidae

Mayr, 1861

02A22494-EBFB-5CB4-A8CA-B4514B95C41A

##### Note.

In the Nearctic region, the genus *Colobopsis* Mayr, 1861 comprises ~ 7 valid species (Bolton 2025).

##### Diagnosis.

**Figure 21.**
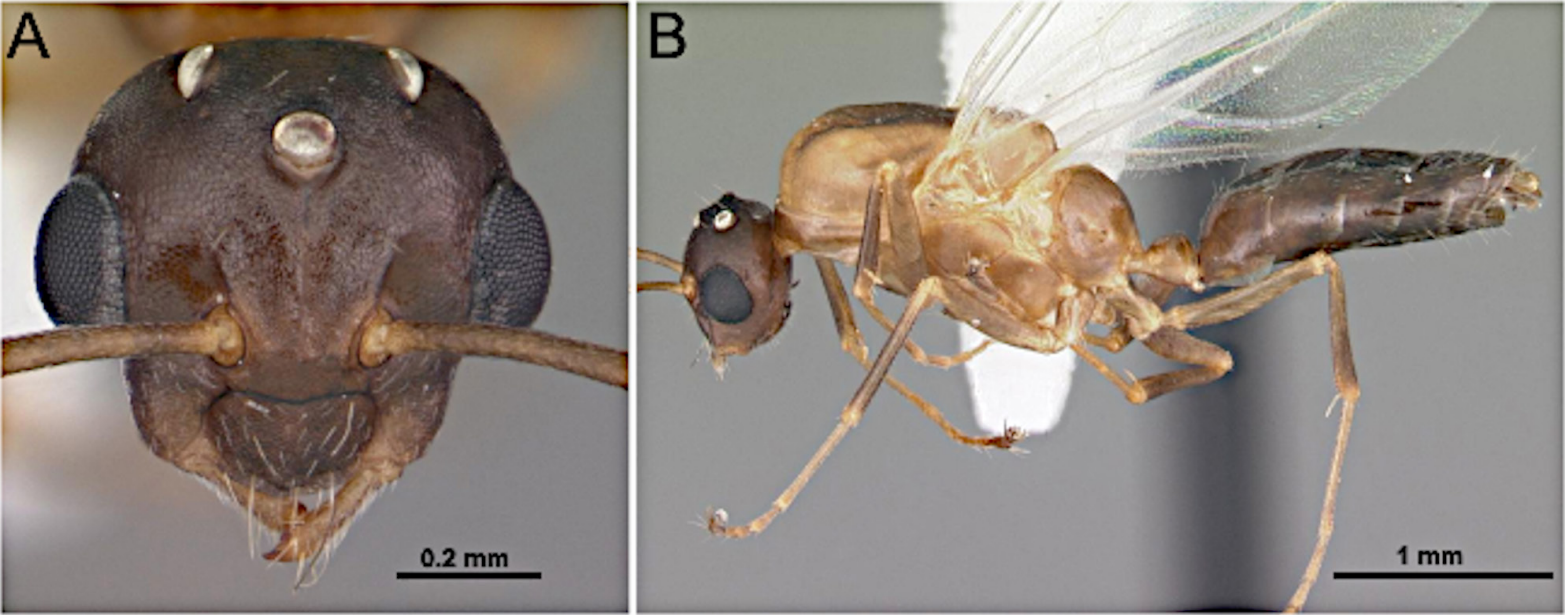
Head in full-face view (**A**) and body in lateral view (**B**) of *Colobopsis impressa* (CASENT0103681; photos by April Nobile; AntWeb.org).

***Head*** (Fig. 21A). Antenna with 13 segments. Medium-sized, overall length 4–6 mm. Mandible robust, triangular; masticatory margin with two or three blunt teeth, inner mandibular margin slightly curved. Palp formula 6:4; maxillary palp slightly longer than compound eye. Antennal sockets positioned at a considerable distance from the posterior margin of the clypeus. Scape lacking erect macrosetae; short, approx. equal to head length. Funiculus shorter than mesosoma length; first flagellomere conspicuously enlarged. Frons lacking paired coarse macrosetae; with impressed midline from median ocellus to level of antennal insertions. Malar space with elongate lateral margin.

***Mesosoma*** (Fig. 21B). Pronotum with a short oblique face, mesoscutum anteriorly strongly convex with straight dorsal face, mesoscutellum moderately elevated, propodeum with distinct dorsal and declivitous face meeting at a rounded angle.

***Male genitalia***. Parameres elongate, apically narrowed; volsella well developed; aedeagus elongate.

***Wings*** present. ***Forewing*** (Fig. 19C): pterostigma well-developed; costal vein (C) present; fused subcostal and radial veins (Sc+R) present; cross vein (2r-rs) connected with radial sector vein at the midpoint of pterostigma; radial sector-medial vein (Rs+M) absent; radial sector vein (Rs) reaching the costal margin; medial-cubital (M+Cu) vein present; first medial-cubital crossvein (1m-cu) absent; crossvein (2rs-m) present; media vein (M) incomplete, not reaching the wing margin; cubital-anal crossvein (cu-a) proximal to junction between media and cubitus; anal vein (A) longer than the fused medial-cubital vein M+Cu. ***Hindwing*** (Fig. 19D): fused radius and radial sector vein (R+Rs) present; medial-cubital (M+Cu) vein present; fused radial sector-medial crossvein and medial vein (1rs-m+M) present; cubital-anal crossvein (cu-a) present; free section of cubitus (Cu) present; anal vein (A) shorter than M+Cu.

##### Remarks.

Males of *Colobopsis* resemble those of *Camponotus* but are typically smaller and more compact, relatively with short scape. In contrast to *Camponotus*, scape usually extend only slightly beyond the posterior margin of the head. Although males lack the phragmotic head modifications of major workers, the genus is generally recognizable by the combination of a short scape and compact mesosoma.

##### Biology and distribution.

Species of *Colobopsis* are primarily arboreal, nesting in pre-existing cavities in twigs, branches, and dead wood. Colonies are generally small and often occur in forested habitats where suitable nesting sites are available. Many species exhibit marked worker polymorphism, with phragmotic major workers bearing a strongly truncated head used to block nest entrances. Workers typically forage on vegetation and tree trunks, collecting honeydew from hemipterans and occasionally preying on small arthropods. In the Nearctic region, *Colobopsis* species occur mainly in temperate and subtropical forests, particularly in the eastern and southern United States (Bolton 2025).

### Tribe Formicini

#### Formica

Taxon classification

Animalia

HymenopteraFormicidae

Linnaeus, 1758

5D1CEA53-9FD4-5D9C-A02F-408988133C61

##### Note.

In the Nearctic region, the genus *Formica* Linnaeus, 1758 includes ~ 106 valid species (Bolton 2025).

##### Diagnosis.

***Head*** (Fig. 23A). Antenna with 12 segments. Medium to large-sized, overall length 4–8 mm. Mandible well developed, triangular, and dentate; masticatory margin with two distinct teeth, with a smaller tooth occasionally present at the basal angle. A large gap occurs between the anterior clypeal margin and the inner mandibular margin, which is curved. Palp formula 6:4 or 5:4; maxillary palp approx. as long as or longer than the maximum eye diameter. Antennal sockets confluent with the posterior edge of the clypeus. Scape lacking erect macrosetae, distinctly longer than the head length. Funiculus approx. equal to or longer than mesosoma length; first flagellomere not enlarged. Frons lacking paired coarse macrosetae; with impressed midline from median ocellus to level of antennal insertions. Malar space with lateral margin prominent.

**Figure 22.**
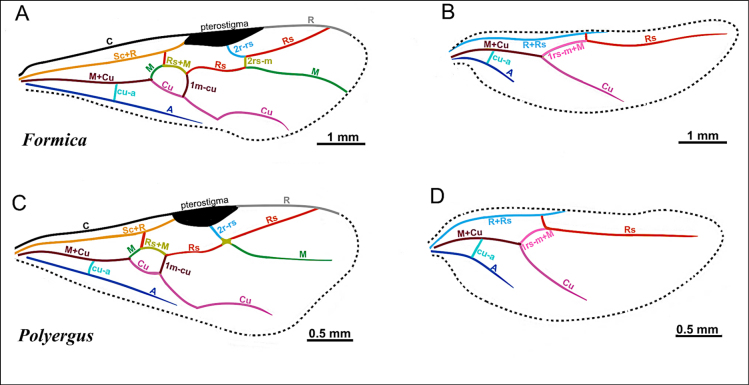
Illustration of fore- and hindwing venation in the tribe Formicini, represented by *Formica* and *Polyergus*, adapted from images (*Formica subaenescens*CASENT0843926, forewing of *Polyergus breviceps*CASENT0100665, hindwing of *Polyergus vinosus*CASENT0281081).

**Figure 23.**
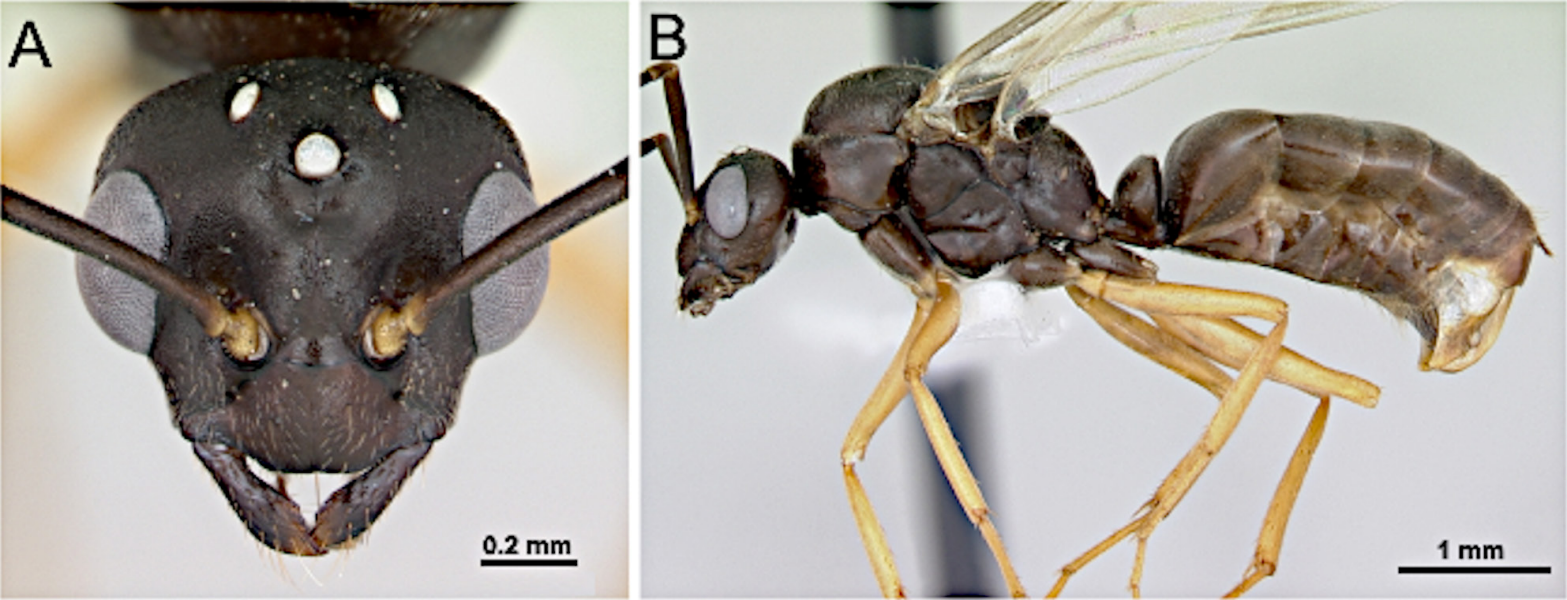
Head in full-face view (**A**) and body in lateral view (**B**) of *Formica wheeleri* (CASENT0173022; photos by April Nobile; AntWeb.org).

***Mesosoma*** (Fig. 23B). Anterior margin of pronotum vertical, mesoscutum smoothly arcuate, mesoscutellum convex; propodeum reduced, dorsum short but distinct, merging gradually with the declivitous face.

***Male genitalia***. Parameres elongate, apically narrow; volsella well developed; aedeagus elongate, weakly curved.

***Wings*** present. ***Forewing***: pterostigma well-developed; costal vein (C) present; fused subcostal and radial veins (Sc+R) present; cross vein (2r-rs) connected with radial sector vein at the midpoint of pterostigma; radial sector-medial vein (Rs+M) present and completely fused with Rs; radial sector vein (Rs) reaching the costal margin; medial-cubital (M+Cu) vein present; first medial-cubital crossvein (1m-cu) present; crossvein (2rs-m) present; media vein (M) incomplete, not reaching the wing margin; cubital-anal crossvein (cu-a) proximal to junction between media and cubitus; anal vein (A) longer than the fused medial-cubital vein M+Cu and Rs+M combined. ***Hindwing***: fused radius and radial sector vein (R+Rs) present; medial-cubital (M+Cu) vein present; fused radial sector-medial crossvein and medial vein (1rs-m+M) present; cubital-anal crossvein (cu-a) present; free section of cubitus (Cu) present; anal vein (A) shorter than M+Cu.

##### Remarks.

Males of *Formica* are clearly recognized by their robust body form, scape elongate, pale tibiae, and well-developed wing venation. They may resemble males of *Polyergus*, but differ in possessing triangular mandible and less reduced wing venation. In comparison with *Camponotus*, males of *Formica* have the head broader posteriorly, the antenna composed of 12 segments (vs 13 in *Camponotus*), and relatively larger eyes.

##### Biology and distribution.

Species of *Formica* are widely distributed across the Nearctic region and are particularly diverse in northern and montane areas of North America. They occupy a broad range of habitats, including forests, grasslands, meadows, and alpine environments (AntWeb 2025). Colonies typically nest in soil or construct conspicuous mound nests composed of soil and plant material. Many species exhibit complex social systems, including temporary social parasitism and dulotic (slave-making) behavior. Males are produced seasonally and are most often encountered during nuptial flights or attracted to lights, and are frequently collected independently of workers in light traps or Malaise traps.

#### Polyergus

Taxon classification

Animalia

HymenopteraFormicidae

Latreille, 1804

2E905F88-1203-526B-BC6D-F0F215136664

##### Note.

In the Nearctic region, the genus *Polyergus* Latreille, 1804 comprises approximately 12 valid species (Bolton 2025).

##### Diagnosis.

**Figure 24.**
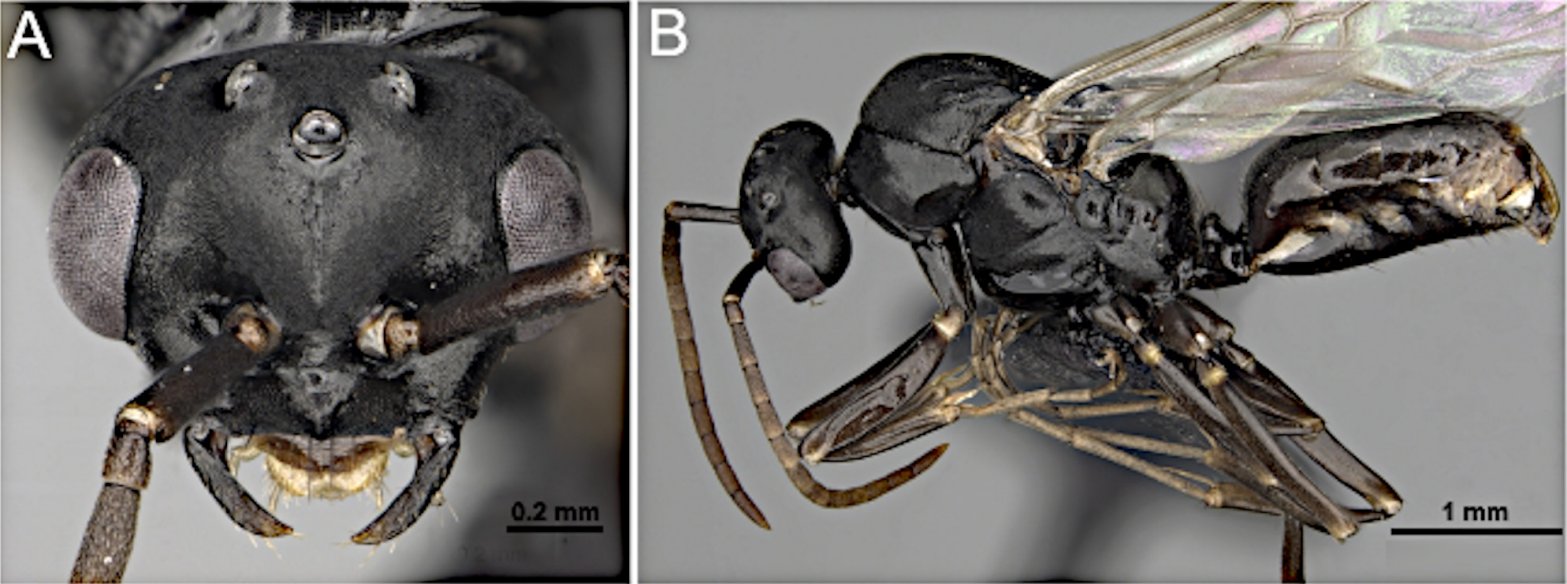
Head in full-face view (**A**) and body in lateral view (**B**) of *Polyergus mexicanus* (CASENT0281072; photos by Shannon Hartman; AntWeb.org).

***Head*** (Fig. 24A). Antenna with 12 segments. Medium-sized, overall length 4.5–6 mm. Mandible reduced, falcate, and denticulate, with the inner margin set far from the anterior clypeal margin. Palp formula: 4:2 or 4:3. Antennal sockets confluent with the clypeus. Scape lacking erect macrosetae; not reaching the posterior margin of the head. Funiculus shorter than mesosoma; first flagellomere not enlarged. Frons lacking paired coarse macrosetae. Malar space laterally well developed.

***Mesosoma*** (Fig. 24B). Dorsal margin of pronotum slightly oblique and forms a right angle with its lateral margin; anterior margin of mesoscutum rounding and meeting the flat dorsal margin; mesoscutellum domelike; propodeum with distinct dorsal and declivitous face.

***Male genitalia***. Parameres elongate, apically narrow; volsella well developed; aedeagus elongate.

***Wings*** present. ***Forewing*** (Fig. 22C): pterostigma well developed; costal vein (C) present; fused subcostal and radial veins (Sc+R) present; cross vein (2r-rs) connected with radial sector vein at the midpoint of pterostigma; radial sector-medial vein (Rs+M) present and completely fused with Rs; radial sector vein (Rs) reaching the costal margin; medial-cubital (M+Cu) vein present; first medial-cubital crossvein (1m-cu) present; crossvein (2rs-m) absent; media vein (M) incomplete, not reaching the wing margin; cubital-anal crossvein (cu-a) proximal to junction between media and cubitus; anal vein (A) longer than the fused medial-cubital vein M+Cu and Rs+M combined. ***Hindwing*** (Fig. 22D): fused radius and radial sector vein (R+Rs) present; medial-cubital (M+Cu) vein present; fused radial sector-medial crossvein and medial vein (1rs-m+M) present; cubital-anal crossvein (cu-a) present; free section of cubitus (Cu) present; anal vein (A) shorter than M+Cu.

##### Remarks.

Males of *Polyergus* are readily recognized by their distinctive mandibular shape and reduced palp formula (4:3 or 4:2). They most closely resemble males of Formica but are generally more slender and differ in mandibular form and wing venation, notably by the absence of the forewing crossvein 2rs–m.

##### Biology and distribution.

In the Nearctic region, the genus *Polyergus* is patchily distributed, with species primarily occurring in the western United States, including California, Arizona, Colorado, Oregon, Washington, and British Columbia. Eastern populations are more scattered, with records from the Midwest, Great Plains, and parts of the Southeast, such as Georgia and Florida. *Polyergus* typically nests in soil, under stones, or in rotting wood, inhabiting a broad range of ecosystems, including coniferous and mixed forests, alpine meadows, prairies, chaparrals, riparian zones, and sandy or rocky woodland habitats. Their presence is closely tied to the availability of Formica hosts, with which they engage in obligate dulotic behavior, often conducting brood raids during the summer months.

### Tribe Lasiini

#### Lasius

Taxon classification

Animalia

HymenopteraFormicidae

Fabricius, 1804

A40AC8DA-DB1A-56DA-9EEB-D6AB21DF7E9D

##### Note.

In the Nearctic region, the genus *Lasius* Fabricius, 1804 comprises ~ 39 valid species (Bolton 2025).

##### Diagnosis.

***Head*** (Fig. 26A). Antenna with 12 segments. Medium-sized, overall length 3.5–6 mm. Mandible noticeably enlarged; masticatory margin edentate or with one tooth; inner margin of mandible with a distinct lateral angle. Palp formula 6:4 or 3:4; maxillary palp exceeding hypostomal margin, exceeding occipital foramen or not. Antennal sockets inserted directly at the posterior clypeal edge. Scape with or without erect macrosetae, shorter than or approx. equal to head length. Funiculus shorter than mesosomal length; first flagellomere shorter and wider than second flagellomere in lateral view. Frons lacking paired coarse macrosetae. Malar space with lateral margin longer than maximum scape width.

**Figure 25.**
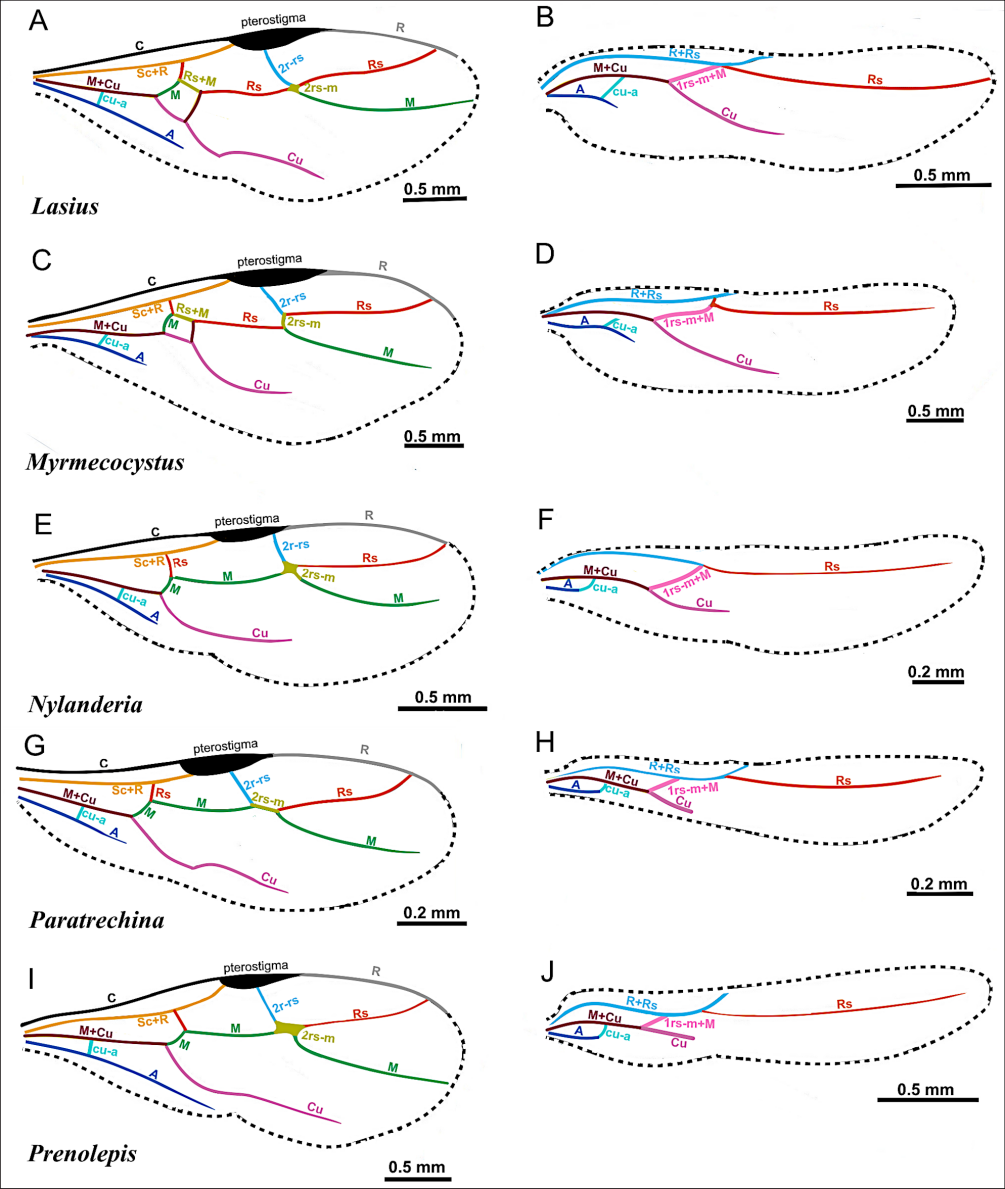
Illustration of fore- and hindwing venation in the tribe Lasiini, represented by *Lasius*, *Myrmecocystus*, *Nylanderia*, *Paratrechina*, *Prenolepis*, adapted from Antweb images (*Lasius neoniger*CASENT0843998, *Myrmecocystus wheeleri*CASENT0862007, *Nylanderia amblyops*CASENT0740912, *Paratrechina longicornis*CASENT0740916, *Prenolepis imparis*: forewing of specimen CASENT0770620, hindwing of specimen CASENT0770599).

**Figure 26.**
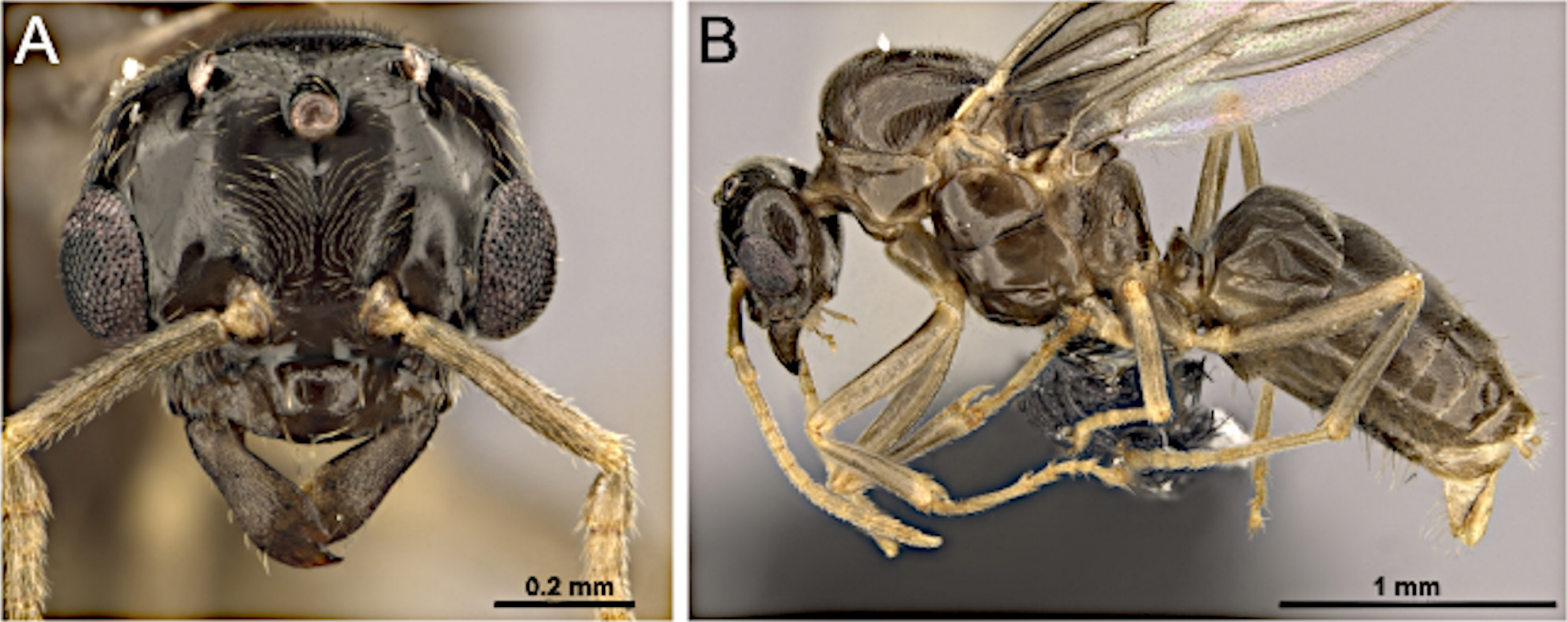
Head in full-face view (**A**) and body in lateral view (**B**) of *Lasius brevicornis* (CASENT0260136; photos by Z. Lieberman; AntWeb.org).

***Mesosoma*** (Fig. 26B). Dorsal margin of pronotum almost vertical, anterior margin of mesoscutum rounding gradually to its dorsal margin, mesoscutellum distinctly convex, propodeum dorsum short and meeting the declivitous face at an obtuse angle.

***Male genitalia***. Parameres short to moderately elongate, apically rounded; volsella compact; aedeagus short.

***Wings*** present. ***Forewing*** (Fig. 25A): pterostigma well developed; costal vein (C) present; fused subcostal and radial veins (Sc+R) present; cross vein (2r-rs) connected with radial sector vein at the midpoint of pterostigma; radial sector-medial vein (Rs+M) present and completely fused with Rs; radial sector vein (Rs) reaching the costal margin; medial-cubital (M+Cu) vein present; first medial-cubital crossvein (1m-cu) present; crossvein (2rs-m) present; media vein (M) incomplete, not reaching the wing margin; cubital-anal crossvein (cu-a) proximal to junction between media and cubitus; anal vein (A) longer than the fused medial-cubital vein M+Cu. ***Hindwing*** (Fig. 25B): fused radius and radial sector vein (R+Rs) present; medial-cubital (M+Cu) vein present; fused radial sector-medial crossvein and medial vein (1rs-m+M) present; cubital-anal crossvein (cu-a) present; free section of cubitus (Cu) present; anal vein (A) shorter than M+Cu.

##### Remarks.

Males of *Lasius* resemble those of *Nylanderia* but can be distinguished by the combination of the following characters: scape lacking erect macrosetae, funiculus shorter than mesosoma, a relatively compact mesosoma, wings relatively long and broad relative to body size, and a petiole forming a high, upright squamiform node. The propodeal dorsum is short, ~ 1/3 the length of the declivitous face.

##### Biology and distribution.

Species of *Lasius* are widely distributed across the Nearctic region and occur throughout much of North America, from the Pacific Coast (e.g., California, Washington, Oregon) to the Atlantic seaboard (e.g., New York, Florida, Massachusetts), with some species extending into northern Mexico. Colonies typically nest in soil, beneath stones, or in decaying wood, and many species maintain trophobiotic associations with honeydew-producing hemipterans.

#### Myrmecocystus

Taxon classification

Animalia

HymenopteraFormicidae

Wesmael, 1838

BCDE0164-0497-5BB9-9D77-AB98A71401E9

##### Note.

In the Nearctic region, the genus *Myrmecocystus* Wesmael, 1838 comprises approximately 30 valid species (Bolton 2025).

##### Diagnosis.

**Figure 27.**
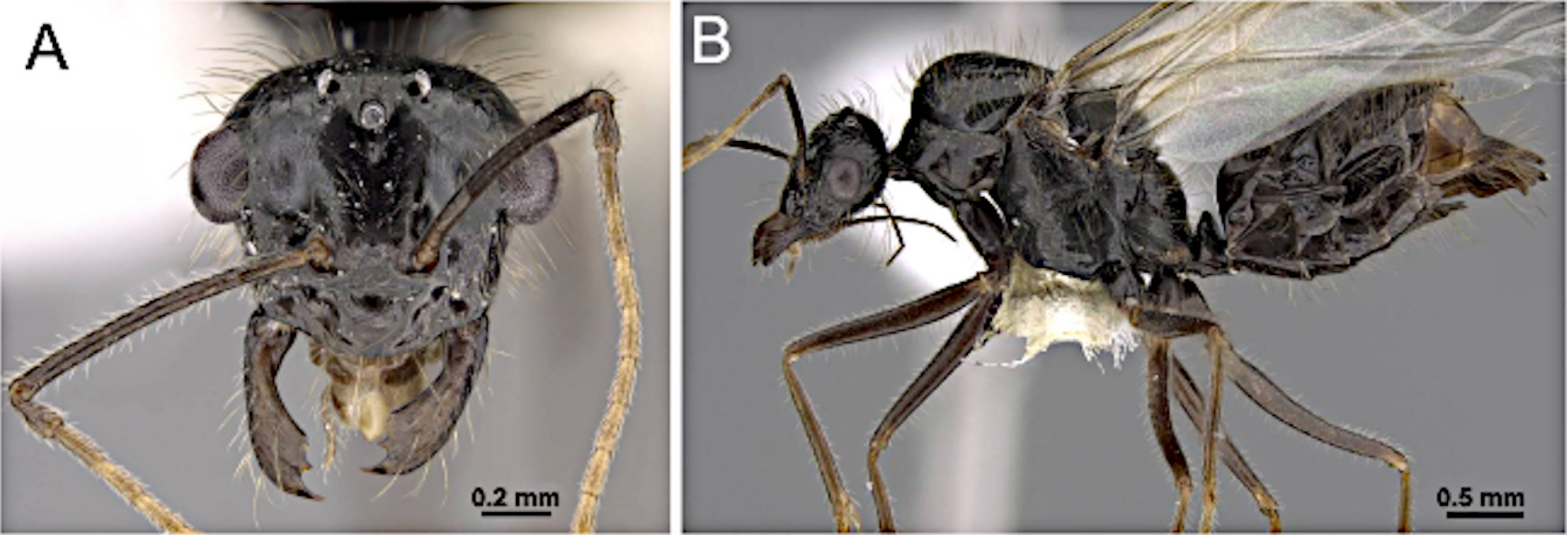
Head in full-face view (**A**) and body in lateral view (**B**) of *Myrmecocystus kathjuli* (CASENT0862007; photos by Michele Esposito; AntWeb.org).

***Head*** (Fig. 27A). Antenna with 12 segments. Medium to large-sized, overall length 5–9 mm. Mandible thickened, subtriangular; masticatory margin with two distinct teeth, a smaller tooth occasionally present at basal angle; inner mandibular margin close to anterior clypeal margin. Palp formula 6:4; maxillary palp greatly elongated, with individual segments slender and cylindrical. Antennal socket confluent with the posterior margin of the clypeus. Scape lacking erect macrosetae, clearly surpassing the posterior margin by approximately the length of the first two flagellomeres. Funiculus shorter than mesosoma length; first flagellomere ~ 2 × the length of the second flagellomere in medial view. Frons lacking paired coarse macrosetae. Malar space laterally well developed.

***Mesosoma*** (Fig. 27B). Dorsal margin of pronotum slightly concave anteriorly; anterior margin of mesoscutum meeting dorsal margin at a rounded angle; mesoscutellum weakly convex; propodeal dorsum greatly reduced, merging with the declivitous face, the latter straight to feebly convex.

***Male genitalia***. Parameres elongate, apically tapered; volsella well developed; aedeagus elongate.

***Wings*** consistently present. ***Forewing*** (Fig. 25C): pterostigma well-developed; costal vein (C) present; fused subcostal and radial veins (Sc+R) present; cross vein (2r-rs) connected with radial sector vein at the midpoint of pterostigma; radial sector-medial vein (Rs+M) present and completely fused with Rs; radial sector vein (Rs) reaching the costal margin; medial-cubital (M+Cu) vein present; first medial-cubital crossvein (1m-cu) present; crossvein (2rs-m) present; media vein (M) incomplete, not reaching the wing margin; cubital-anal crossvein (cu-a) proximal to junction between media and cubitus; anal vein (A) shorter or subequal to the fused medial-cubital vein M+Cu. ***Hindwing*** (Fig. 25D): fused radius and radial sector vein (R+Rs) present; medial-cubital (M+Cu) vein present; fused radial sector-medial crossvein and medial vein (1rs-m+M) present; cubital-anal crossvein (cu-a) present; free section of cubitus (Cu) present; anal vein (A) shorter than M+Cu.

##### Remarks.

Males of *Myrmecocystus* in the Nearctic region are recognized by the combination of elongate maxillary palps (palp formula 6:4), scape clearly surpassing the posterior margin of the head, subtriangular mandible with two principal teeth, and a psammophore on the ventral surface of the head.

##### Biology and distribution.

Species of *Myrmecocystus* occur primarily in arid and semi-arid regions of western North America, with records extending from California and Arizona to Utah and Texas, and locally into the Pacific Northwest and parts of the Midwest. Commonly known as “honeypot ants,” these species inhabit desert ecosystems as well as pinyon–juniper woodlands and chaparral. Colonies typically nest in soil, often beneath shrubs such as creosote bush, and many species possess specialized replete workers that store liquid food as a reserve for the colony.

#### Nylanderia

Taxon classification

Animalia

HymenopteraFormicidae

Emery, 1906

0CAD42CD-199C-5242-A0F1-CAECC08434A4

##### Note.

In the Nearctic region, the genus *Nylanderia* Emery, 1906 comprises ~ 30 valid species (Bolton 2025).

##### Diagnosis.

**Figure 28.**
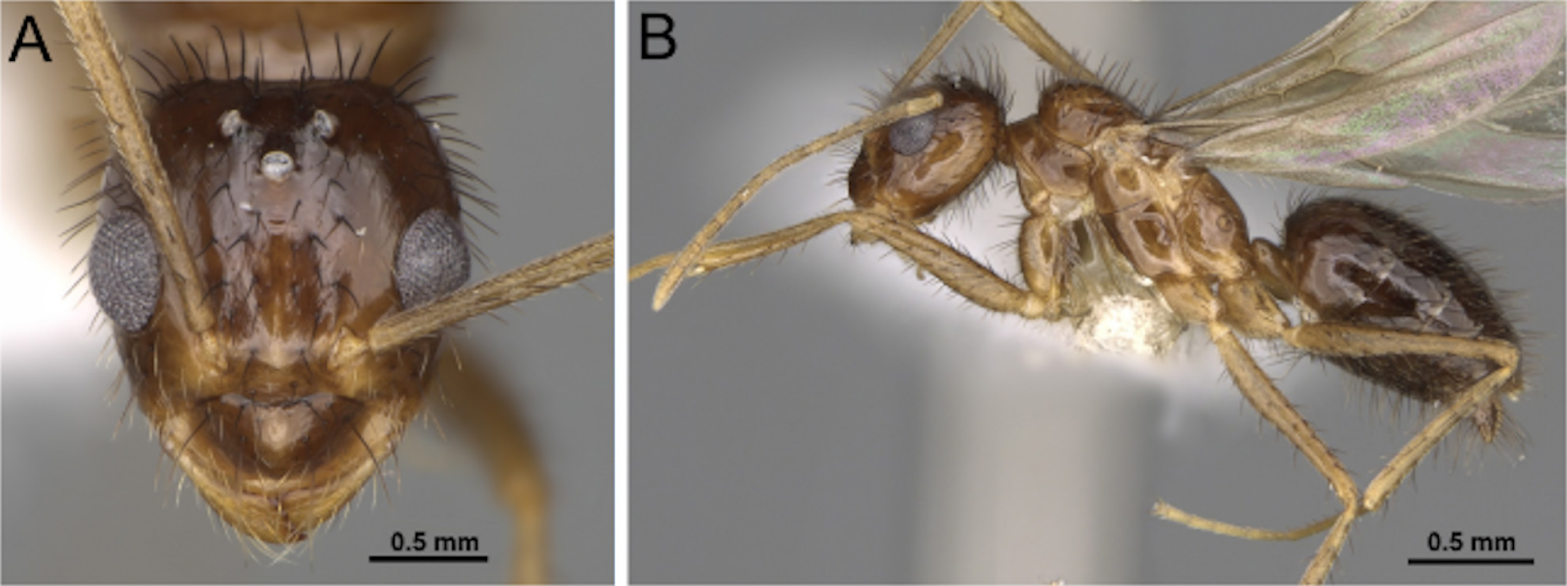
Head in full-face view (**A**) and body in lateral view (**B**) of *Nylanderia hystrix* (CASENT0106724; photo by Michele Esposito; AntWeb.org).

***Head*** (Fig. 28A). Antenna with 12–13 segments. Small to medium-sized, overall length 2.5–4.5 mm. Mandible broad, triangular; masticatory margin with two denticles. Palp formula 6:4; maxillary palp longer than compound eye diameter and shorter than head length. Antennal insertions confluent with the posterior clypeal border. Scape usually with erect macrosetae, longer than head length but distinctly shorter than mesosoma length. Funiculus longer than mesosoma length; first flagellomere distinctly longer than second flagellomere in medial view. Frons with paired coarse macrosetae. Malar space laterally extended, approximately equal to the length of the first flagellomere.

***Mesosoma*** (Fig. 28B). Dorsal margin of pronotum slightly oblique; anterior margin of mesoscutum meeting dorsal margin at a rounded angle; mesoscutellum almost straight; propodeal dorsum longer than declivitous face, meeting at a rounded angle.

***Male genitalia***. Parameres short to moderately elongate, apically rounded or weakly digitiform; volsella compact; aedeagus short.

***Wings*** present or absent. ***Forewing*** (Fig. 25E): pterostigma well-developed; costal vein (C) present; fused subcostal and radial veins (Sc+R) present; cross vein (2r-rs) connected with radial sector vein at the endpoint of pterostigma; radial sector-medial vein (Rs+M) absent; radial sector vein (Rs) reaching the costal margin; medial-cubital (M+Cu) vein present; first medial-cubital crossvein (1m-cu) absent; crossvein (2rs-m) present; media vein (M) incomplete, not reaching the wing margin; cubital-anal crossvein (cu-a) proximal to junction between media and cubitus; anal vein (A) shorter or subequal to the fused medial-cubital vein M+Cu. ***Hindwing*** (Fig. 25F): fused radius and radial sector vein (R+Rs) present; medial-cubital (M+Cu) vein present; fused radial sector-medial crossvein and medial vein (1rs-m+M) present; cubital-anal crossvein (cu-a) present; free section of cubitus (Cu) present; anal vein (A) shorter, not surpassing the junction with the cu-a vein.

Males of the following *Nylanderia* species are recorded as wingless in the Nearctic region: *Nylanderia parasitica* Messer, Cover & Rabeling, 2020, *Nylanderia deyrupi* Messer, Cover & Rabeling, 2020, *Nylanderia deceptrix* Messer, Cover & LaPolla, 2016.

##### Remarks.

Males of *Nylanderia* are recognized by the presence of erect macrosetae on the scape and frons. They most closely resemble males of *Paratrechina* but lack the extremely elongate appendages characteristic of that genus; the antenna and legs are comparatively shorter and more robust.

##### Biology and distribution.

Species of *Nylanderia* are widely distributed across the United States, occurring from the Southeast (e.g., Florida, Georgia, South Carolina, Alabama, Mississippi) and Midwest (e.g., Illinois, Indiana, Ohio, Iowa, Missouri) to the Northeast (e.g., New York, New Jersey, Massachusetts, Pennsylvania, Maryland, District of Columbia) and western states (e.g., California, Arizona, Nevada, Utah, Colorado, Washington). Members of the genus occupy a broad range of habitats, including pine–hardwood forests, bottomland hardwoods, sandhills, prairies, coastal scrub, and desert riparian zones, and are also common in urban and suburban environments (AntWeb 2025). Colonies typically nest in soil and leaf litter, beneath logs or stones, and within decaying wood.

#### Paratrechina

Taxon classification

Animalia

HymenopteraFormicidae

Motschoulsky, 1863

6CC7318B-886D-5EC4-90F8-6DB70C1A8D5A

##### Note.

In the Nearctic region, the genus *Paratrechina* Motschoulsky, 1863 is represented by the cosmopolitan species *Paratrechina longicornis* (Latreille, 1802) (Bolton 2025).

#### Paratrechina longicornis

Taxon classification

Animalia

HymenopteraFormicidae

Latreille, 1802

64B93E67-FCCB-5019-811C-E049B40E78A7

**Figure 29.**
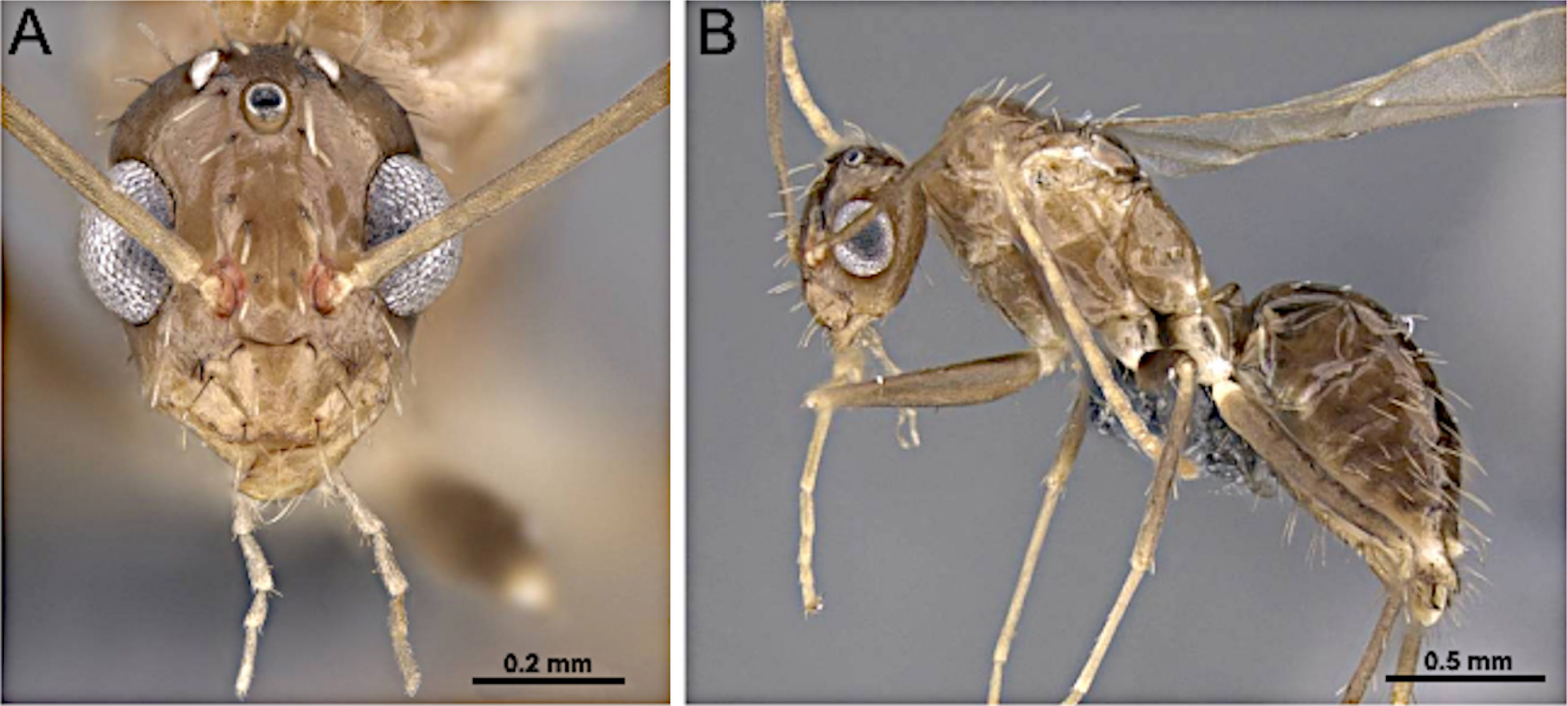
Head in full-face view (**A**) and body in lateral view (**B**) of *Paratrechina longicornis* (CASENT0740916; photos by Veronica M. Sinotte; AntWeb.org).

Figs 1D, 9A, 13A, 13B, 29A, 29B

##### Diagnosis.

***Head*** (Fig. 29A). Antenna with 13 segments. Medium-sized, overall length 3.5–5.5 mm. Mandible moderately broad, spatulate; masticatory margin with single apical tooth. Palp formula 6:4; maxillary palp as long as head. Antennal sockets contiguous with the posterior margin of the clypeus. Scape lacking erect macrosetae; extremely long. Funiculus longer than mesosoma length; first flagellomere slightly shorter than the second flagellomere in medial view. Frons with paired coarse macrosetae. Malar space with an extended lateral border.

***Mesosoma*** (Fig. 29B). Dorsal margin of pronotum slightly oblique; anterior margin of mesoscutum meeting dorsal margin at a rounded angle; mesoscutellum slightly convex; propodeal dorsum smoothly curved into declivitous face; propodeal angle effaced.

***Male genitalia***. Parameres elongate, apically rounded to weakly tapered; volsella well developed; aedeagus elongate.

***Wings*** consistently present. ***Forewing*** (Fig. 25G): pterostigma well developed; costal vein (C) present; fused subcostal and radial veins (Sc+R) present; cross vein (2r-rs) connected with radial sector vein at the midpoint of pterostigma; radial sector-medial vein (Rs+M) absent; radial sector vein (Rs) reaching the costal margin; medial-cubital (M+Cu) vein present; first medial-cubital crossvein (1m-cu) absent; crossvein (2rs-m) present; media vein (M) incomplete, not reaching the wing margin; cubital-anal crossvein (cu-a) proximal to junction between media and cubitus; anal vein (A) shorter or subequal to the fused medial-cubital vein M+Cu. ***Hindwing*** (Fig. 25H): fused radius and radial sector vein (R+Rs) present; medial-cubital (M+Cu) vein present; fused radial sector-medial crossvein and medial vein (1rs-m+M) present; cubital-anal crossvein (cu-a) present; free section of cubitus (Cu) present; anal vein (A) shorter, not surpassing the junction with the cu-a vein.

##### Remarks.

Males of *Paratrechina longicornis* are distinguished from *Nylanderia* by their extremely elongate scape lacking erect macrosetae and strongly surpassing the posterior head margin (scape shorter and with erect macrosetae in *Nylanderia*). They further differ by the presence of a single apical mandibular tooth, maxillary palps as long as the head, and a funiculus longer than the mesosoma.

##### Biology and distribution.

*Paratrechina longicornis* exhibits broad ecological tolerance and occurs in a wide range of natural and anthropogenic habitats. In the Nearctic region, it is established outdoors across the southeastern United States from South Carolina to Florida and west to Texas, and also occurs in California and other southern states; farther north it is typically confined to indoor environments. Colonies nest in soil, debris, and structural cavities, and workers commonly forage in disturbed and urban habitats, reflecting the strongly synanthropic habits of this cosmopolitan tramp species.

#### Prenolepis

Taxon classification

Animalia

HymenopteraFormicidae

Mayr, 1861

196DB825-F8A8-5D9A-8E9F-1DB4EDD0601C

##### Note.

In the Nearctic region, the genus *Prenolepis* Mayr, 1861 is represented by a single valid species, *Prenolepis imparis* (Say, 1836) (Bolton 2025).

#### Prenolepis imparis

Taxon classification

Animalia

HymenopteraFormicidae

Say, 1836

4BFD06D7-E75A-5033-A3DD-273C3D2B6EFC

**Figure 30.**
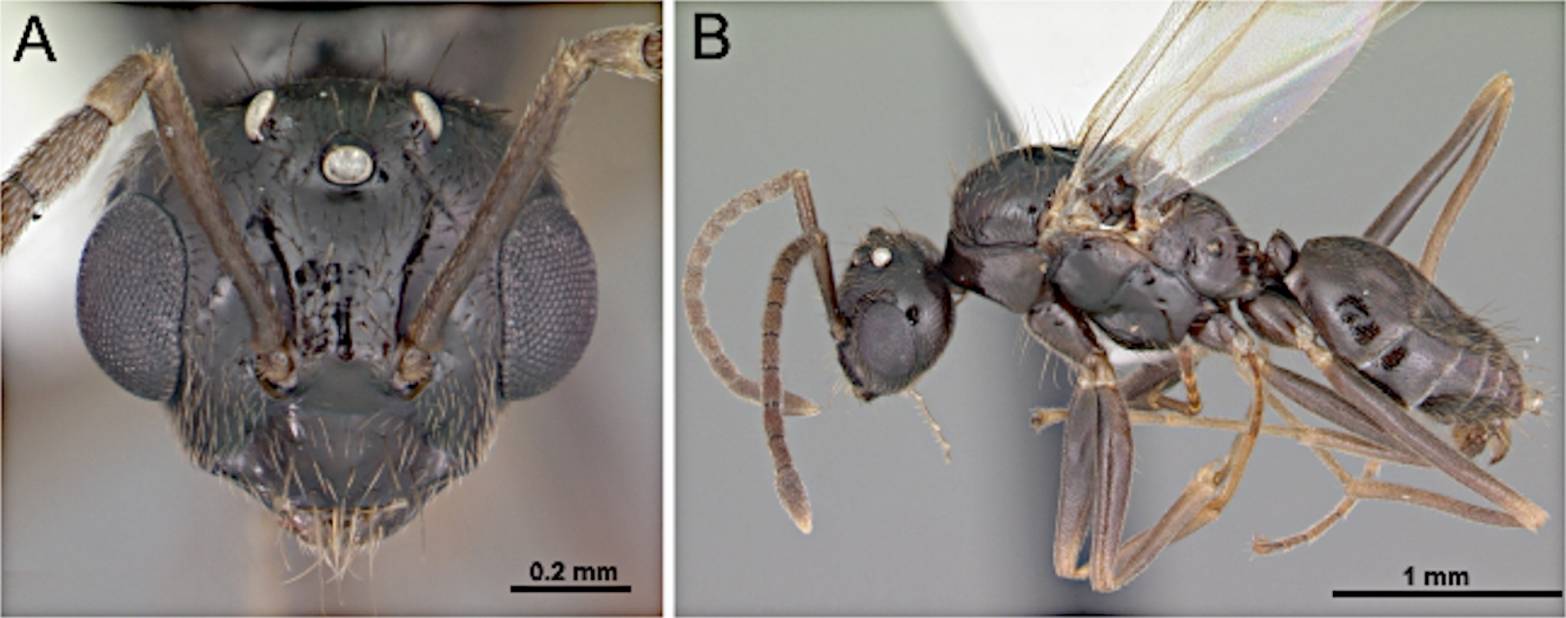
Head in full-face view (**A**) and body in lateral view (**B**) of *Prenolepis imparis* (CASENT0104433; photos by April Nobile; AntWeb.org).

Figs 10A, 10B, 30A, 30B

##### Diagnosis.

***Head*** (Fig. 30A). Antenna with 13 segments. Medium-sized, overall length 4–6 mm. Mandible well developed; masticatory margin with a single apical tooth. Palp formula 6:4; maxillary palp very long, reaching the occipital margin. Antennal sockets closely approximated to the posterior margin of the clypeus. Scape lacking erect macrosetae; barely surpassing the posterior margin of the head. Funiculus longer than mesosoma length; first flagellomere slightly shorter than second flagellomere in medial view. Frons without paired coarse macrosetae. Malar space laterally long.

***Mesosoma*** (Fig. 30B). Dorsal margin of pronotum inclined posteriorly; anterior margin of mesoscutum rounding and meeting the dorsal margin; mesoscutellum smoothly curved; propodeum evenly rounded, forming a continuous slope without a distinct base and declivity.

***Male genitalia***. Parameres moderately elongate, apically rounded; volsella well developed; aedeagus elongate.

***Wings*** consistently present. ***Forewing*** (Fig. 25I): pterostigma well developed; costal vein (C) present; fused subcostal and radial veins (Sc+R) present; cross vein (2r-rs) connected with radial sector vein at the midpoint of pterostigma; radial sector-medial vein (Rs+M) absent; radial sector vein (Rs) reaching the costal margin; medial-cubital (M+Cu) vein present; first medial-cubital crossvein (1m-cu) absent; crossvein (2rs-m) present; media vein (M) incomplete, not reaching the wing margin; cubital-anal crossvein (cu-a) proximal to junction between media and cubitus; anal vein (A) shorter or subequal to the fused medial-cubital vein M+Cu. ***Hindwing*** (Fig. 25J): fused radius and radial sector vein (R+Rs) present; medial-cubital (M+Cu) vein present; fused radial sector-medial crossvein and medial vein (1rs-m+M) present; cubital-anal crossvein (cu-a) present; free section of cubitus (Cu) present; anal vein (A) shorter, not surpassing the junction with the cu-a vein.

##### Remarks.

Males of *Prenolepis imparis* are recognized by relatively short scape without erect macrosetae and by the large eyes, which are longer than the postocular distance. They most closely resemble *Nylanderia* and *Lasius*, but differ in pilosity pattern and petiolar shape.

##### Biology and distribution.

*Prenolepis imparis* is widely distributed across the United States, with records from numerous states including Alabama, Arizona, California, Colorado, Florida, Georgia, Illinois, Maryland, Michigan, New York, North Carolina, Pennsylvania, Texas, Utah, Virginia, Washington, and Wisconsin. The species occurs in a variety of habitats, ranging from mesic hardwood and mixed forests to more xeric environments such as chaparral and sand pine scrub. Colonies typically nest in soil or beneath stones, logs, and decaying wood, and workers are frequently collected in leaf litter and at the bases of trees. The species also occurs in disturbed environments such as suburban yards, botanical gardens, roadsides, and urban parks (AntWeb 2025).

### Tribe Myrmelachistini

#### Brachymyrmex

Taxon classification

Animalia

HymenopteraFormicidae

Mayr, 1868

C76675C9-6B8F-5909-B14F-E232242AE06D

##### Note.

In the Nearctic region, the genus *Brachymyrmex* Mayr, 1868 comprises approximately 6 valid species (Bolton 2025).

##### Diagnosis.

***Head*** (Fig. 32A). Antenna with ten segments. Males small, overall length 2–2.5 mm. Mandible reduced, spatulate to spiniform; masticatory margin with one or two teeth. Palp formula 5:3; maxillary palp approximately as long as maximum eye diameter. Antennal sockets abutting the posterior margin of the clypeus. Scape lacking erect macrosetae and shorter than head length. Funiculus shorter than mesosoma length; first flagellomere enlarged, slightly longer than second flagellomere in medial view. Frons lacking paired coarse macrosetae. Malar space laterally well developed.

**Figure 31.**
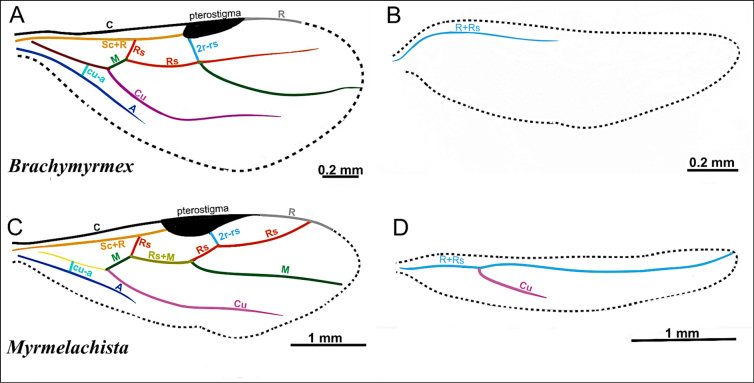
Illustration of fore- and hindwing venation in the tribe Myrmelachistini, represented by *Brachymyrmex* and *Myrmelachista*, adapted from Antweb images (*Brachymyrmex cordemoyi*CASENT0740909, *Myrmelachista catharinae*CASENT0915738).

**Figure 32.**
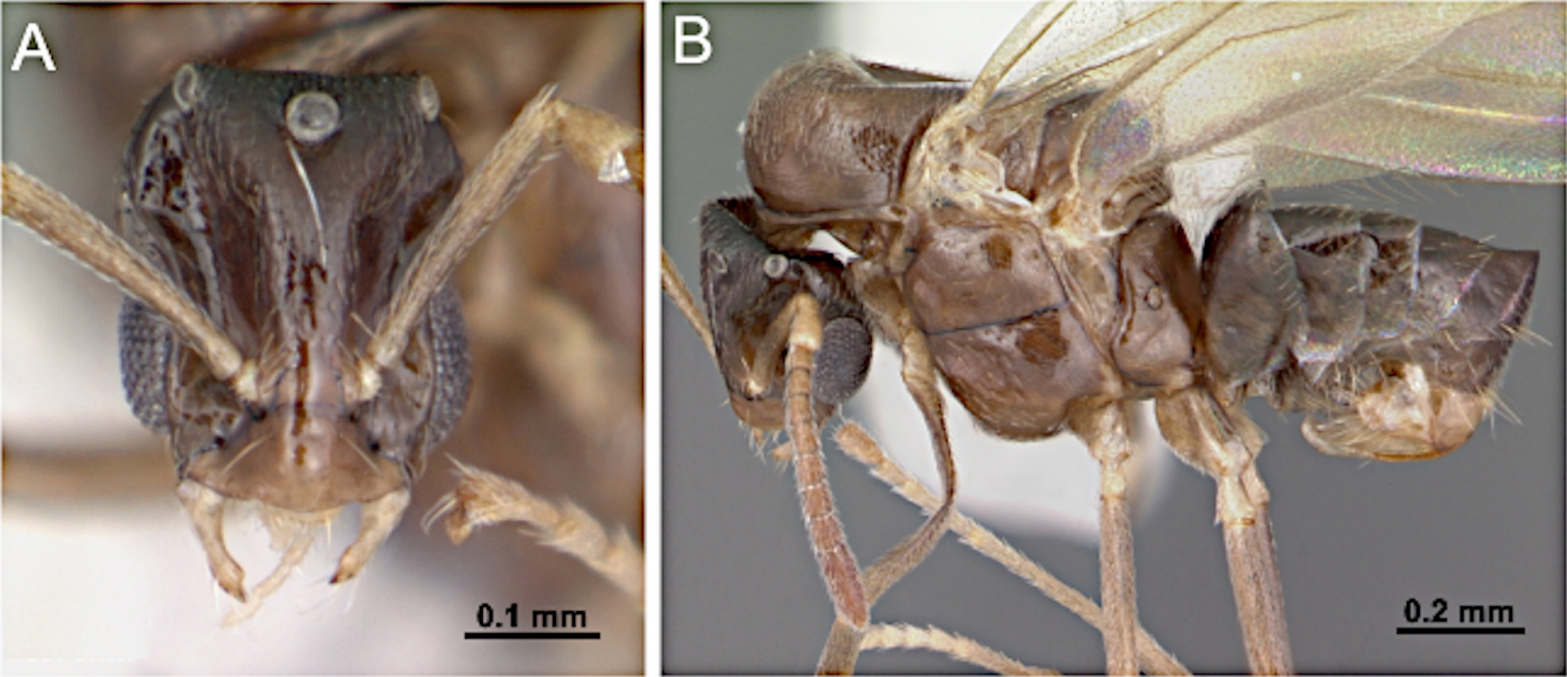
Head in full-face view (**A**) and body in lateral view (**B**) of *Brachymyrmex cf. obscurior* (CASENT0103641; photos by April Nobile; AntWeb.org).

***Mesosoma*** (Fig. 32B). Dorsal margin of pronotum relatively short, anterior margin of mesoscutum prominently anterodorsally produced, with a straight dorsum, mesoscutellum posterodorsally produced, its posterior face aligned with the declivitous face of the propodeum but separated by a weakly impressed metanotal suture; dorsum of propodeum reduced.

***Male genitalia***. Parameres short, apically rounded; volsella compact; aedeagus short and simple.

***Wings*** present. ***Forewing*** (Fig. 31A): pterostigma well developed; costal vein (C) present; fused subcostal and radial veins (Sc+R) present; cross vein (2r-rs) connected with radial sector vein at the basal margin of pterostigma; radial sector-medial vein (Rs+M) absent; radial sector vein (Rs) not reaching the costal margin; medial-cubital (M+Cu) vein present; first medial-cubital crossvein (1m-cu) absent; crossvein (2rs-m) absent; media vein (M) incomplete, not reaching the wing margin; cubital-anal crossvein (cu-a) proximal to junction between media and cubitus; anal vein (A) longer than the fused medial-cubital vein M+Cu. ***Hindwing*** (Fig. 31B): fused radius and radial sector vein (R+Rs) present; medial-cubital (M+Cu) vein absent; fused radial sector-medial crossvein and medial vein (1rs-m+M) absent; cubital-anal crossvein (cu-a) absent; free section of cubitus (Cu) absent; anal vein (A) absent.

##### Remarks.

Males of *Brachymyrmex* are readily recognized among Nearctic formicines by the combination of the following characters: antenna with ten segments, reduced mandible, and palp formula 5:3. They are small and delicate and may resemble *Plagiolepis*, but differ in the reduced number of antennal segments and in the mesoscutum, which is distinctly produced anteriorly and the venation on the hindwing is greatly reduced.

##### Biology and distribution.

Species of *Brachymyrmex* occur in a variety of habitats, including forests, grasslands, coastal savannas, deserts, and urban environments. Colonies typically nest in soil, leaf litter, and decaying wood, and workers are frequently encountered in cryptic microhabitats (AntWeb 2025). In the Nearctic region, the genus is widely distributed across the United States, reflecting its ecological versatility and broad tolerance of environmental conditions.

#### Myrmelachista

Taxon classification

Animalia

HymenopteraFormicidae

Roger, 1863

AE26DD65-D635-546F-8380-A00934C1D7CF

##### Note.

In the Nearctic region, the genus *Myrmelachista* Roger, 1863 is represented by a single valid species, *Myrmelachista ramulorum* (Wheeler, 1904) (Bolton 2025). However, as males of *M. ramulorum* are unknown, males of *Myrmelachista rsp069* and *Myrmelachista catharinae* are used to illustrate the key and accompanying description.

##### Diagnosis.

**Figure 33.**
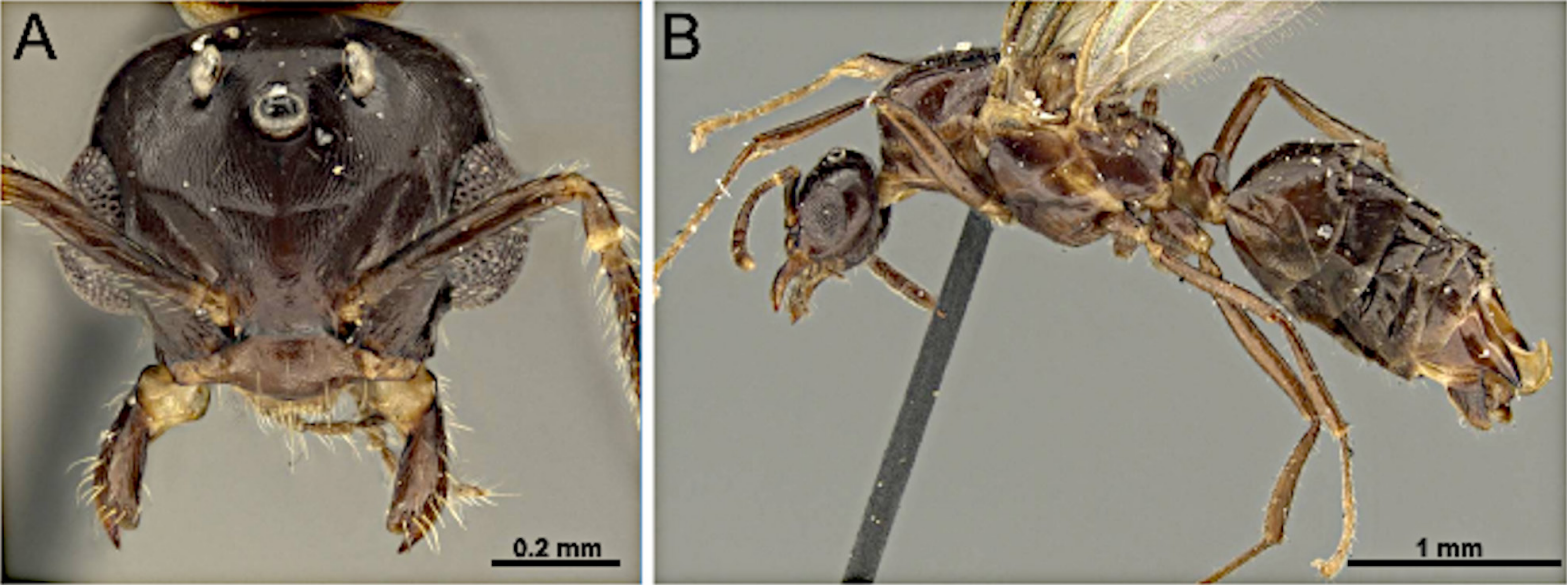
Head in full-face view (**A**) and body in lateral view (**B**) of *Myrmelachista catharinae* (CASENT0915738; photos by Daniela Lehner, AntWeb.org). [Shown here as representative; Nearctic species *M. ramulorum* not illustrated.]

***Head*** (Fig. 33A). Antenna with nine or ten segments. Small to medium-sized, overall length 2.5–4 mm. Mandible moderately developed, subtriangular dentate; masticatory margin with two large teeth. Palp formula 6:4; maxillary palp longer than compound eye diameter and shorter than head length. Antennal sockets inserted close to the posterior edge of the clypeus. Scape lacking erect macrosetae; shorter than head length. Funiculus shorter than the mesosoma; first flagellomere enlarged. Frons lacking paired coarse macrosetae; with an impressed midline extending from the median ocellus to the level of the antennal insertions. Malar space laterally developed.

***Mesosoma*** (Fig. 33B). Dorsal margin of pronotum slightly concave anteriorly; anterior margin of mesoscutum rounding into dorsal margin; mesoscutellum weakly convex; propodeal dorsum distinct, subequal in length to vertical declivitous face.

***Male genitalia***. Parameres short, apically rounded; volsella reduced; aedeagus short and simple.

***Wings*** consistently present. ***Forewing*** (Fig. 31A): pterostigma well developed; costal vein (C) present; fused subcostal and radial veins (Sc+R) present; cross vein (2r-rs) connected with radial sector vein at the midpoint of pterostigma; radial sector-medial vein (Rs+M) present and completely fused with Rs; radial sector vein (Rs) reaching the costal margin; medial-cubital (M+Cu) vein present; first medial-cubital crossvein (1m-cu) absent; crossvein (2rs-m) absent; media vein (M) incomplete, not reaching the wing margin; cubital-anal crossvein (cu-a) proximal to junction between media and cubitus; anal vein (A) longer than the fused medial-cubital vein M+Cu. ***Hindwing*** (Fig. 31B): fused radius and radial sector vein (R+Rs) present; medial-cubital (M+Cu) vein absent; fused radial sector-medial crossvein and medial vein (1rs-m+M) absent; cubital-anal crossvein (cu-a) absent; free section of cubitus (Cu) present; anal vein (A) absent.

##### Remarks.

Males of *Myrmelachista* are distinguished by antenna with 9–10 segments, scape lacking erect macrosetae, and an enlarged first flagellomere. The petiolar node is distinctly nodiform. This combination of characters separates *Myrmelachista* from *Plagiolepis* and *Brachymyrmex*, both of which lack a nodiform petiole.

##### Biology and distribution.

In the Nearctic region, *Myrmelachista ramulorum* has been recorded from Highland City, Florida. This species is considered introduced (Deyrup et al. 2000), but subsequent surveys suggest it may no longer be established in the state (Deyrup 2017).

### Tribe Plagiolepidini

#### Acropyga

Taxon classification

Animalia

HymenopteraFormicidae

Roger, 1862

D1274C19-4BED-5AA5-B878-86E264FF4CC0

##### Note.

In the Nearctic region, the genus *Acropyga* Roger, 1862 is represented by a single valid species, *Acropyga epedana* Wheeler, 1905 (Bolton 2025).

#### Acropyga epedana

Taxon classification

Animalia

HymenopteraFormicidae

Snelling, 1973

2F7265A2-5AE2-58C8-89AC-F5A35ECB2AED

Figs 7A, 7B, 35A, 35B

##### Diagnosis.

***Head*** (Fig. 35A). Antenna with 12 segments. Small-sized, overall length 2.00–2.13 mm. Mandible reduced; masticatory margin with two distinct teeth, a smaller tooth occasionally present at basal angle; large gap exists between anterior clypeal margin and inner mandibular margin which is curved. Palp formula 6:4; maxillary palp slightly longer than compound eye. Antennal sockets positioned at the posterior limit of clypeus. Scape lacking erect macrosetae, surpassing posterior margin by approximately the length of first two flagellomeres. Funiculus shorter than mesosoma; first flagellomere enlarged, slightly longer than second flagellomere. Frons without paired coarse macrosetae. Malar space with an elongate lateral margin.

**Figure 34.**
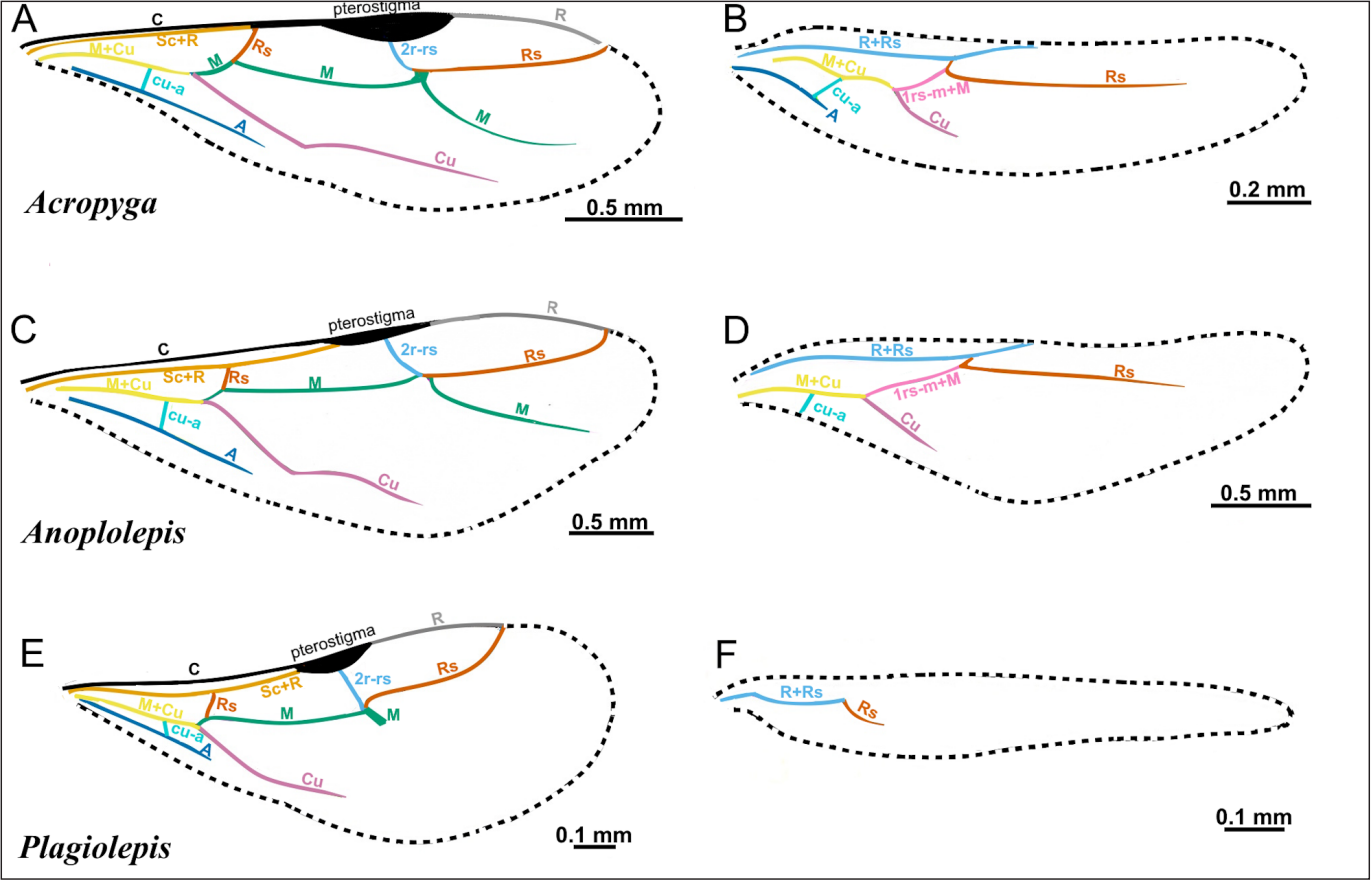
Illustration of fore- and hindwing venation in the tribe Plagiolepidini, represented by *Acropyga*, *Anoplolepis* and *Plagiolepis*, adapted from Antweb images. (*Acropyga myops*CASENT0172017, *Anoplolepis gracilipes*MEM 206500, *Plagiolepis alluaudi*CASENT0495472).

**Figure 35.**
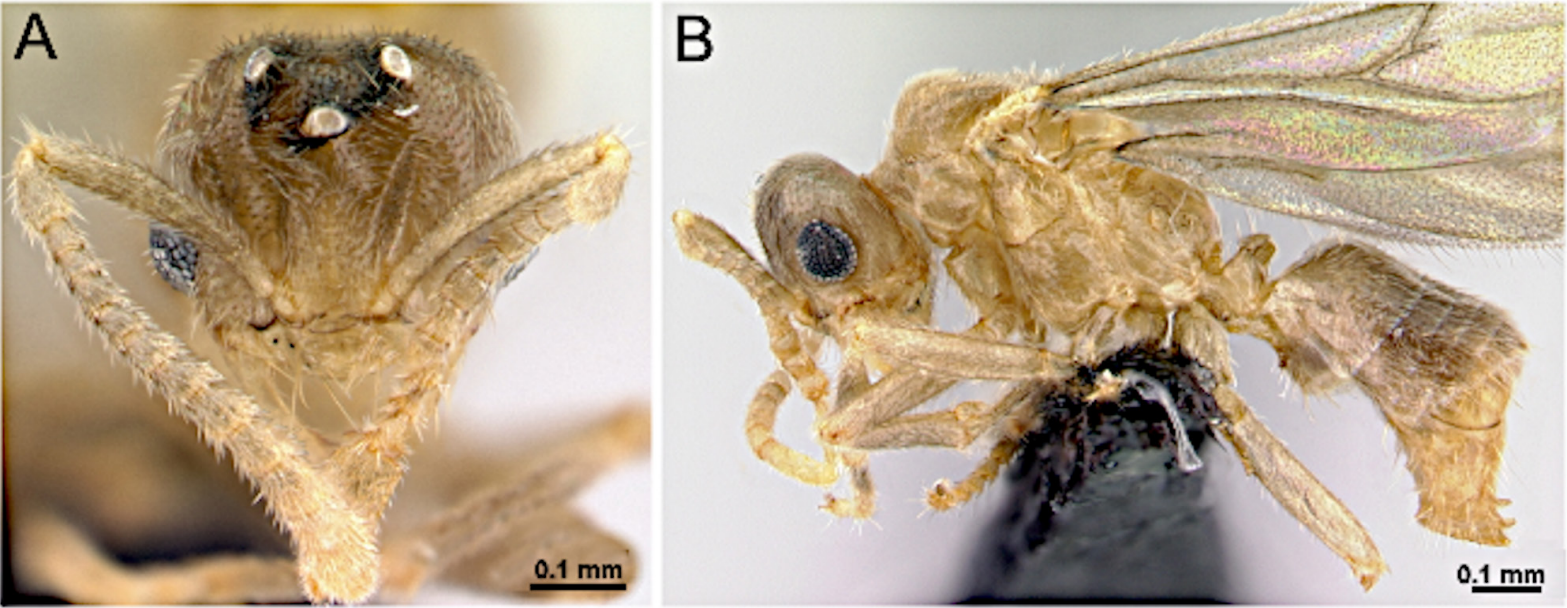
Head in full-face view (**A**) and body in lateral view (**B**) of *Acropyga epedana* (CASENT0188857; photos by Erin Prado, AntWeb.org).

***Mesosoma*** (Fig. 35B). Pronotum with a short, collar-like dorsal margin; anterior margin of mesoscutum gradually rounded, meeting flat dorsal face; dorsal face of mesoscutellum flattened; dorsal face of propodeum short, forming an obtuse angle with short declivitous face.

***Wings*** present. ***Forewing*** (Fig. 34A): pterostigma well developed; costal vein (C) present; fused subcostal and radial veins (Sc+R) present; cross vein (2r-rs) connected with radial sector vein at the midpoint of pterostigma; radial sector-medial vein (Rs+M) absent; radial sector vein (Rs) reaching the costal margin; medial-cubital (M+Cu) vein present; first medial-cubital crossvein (1m-cu) absent; crossvein (2rs-m) absent; media vein (M) incomplete, not reaching the wing margin; cubital-anal crossvein (cu-a) proximal to junction between media and cubitus; anal vein (A) longer than the fused medial-cubital vein M+Cu. ***Hindwing*** (Fig. 34B): fused radius and radial sector vein (R+Rs) present; medial-cubital (M+Cu) vein absent; fused radial sector-medial crossvein and medial vein (1rs-m+M) present; cubital-anal crossvein (cu-a) present; free section of cubitus (Cu) present; anal vein (A) shorter than M+Cu.

##### Remarks.

A detailed description of this species is provided in LaPolla et al. (2002). Males of *Acropyga epedana* are recognized by the dense body pubescence, the relatively large parameres of the genital capsule, and the thick, nodiform petiolar node. These characters distinguish the genus from other small Nearctic formicine genera, such as *Plagiolepis* and *Brachymyrmex*.

##### Biology and distribution.

*Acropyga epedana* is a cryptobiotic species that nests in soil and maintains obligate trophobiotic associations with subterranean mealybugs. Males are rarely collected but may occur in woodland habitats. Specimens from Arizona have been recorded in oak, oak–pine, and oak–pine–juniper woodlands, where individuals were recovered from soil samples, beneath stones, and from ground nests.

#### Anoplolepis

Taxon classification

Animalia

HymenopteraFormicidae

Santschi, 1914

FA87E377-1F8F-59B5-8E01-9869D5834E80

##### Note.

In the Nearctic region, the genus *Anoplolepis* Santschi, 1914 is represented by the introduced species *Anoplolepis gracilipes* (Smith, F., 1857) (Bolton 2025).

#### Anoplolepis gracilipes

Taxon classification

Animalia

HymenopteraFormicidae

Smith, F., 1857

64640F01-5894-5B7A-976E-D209AD4BC325

**Figure 36.**
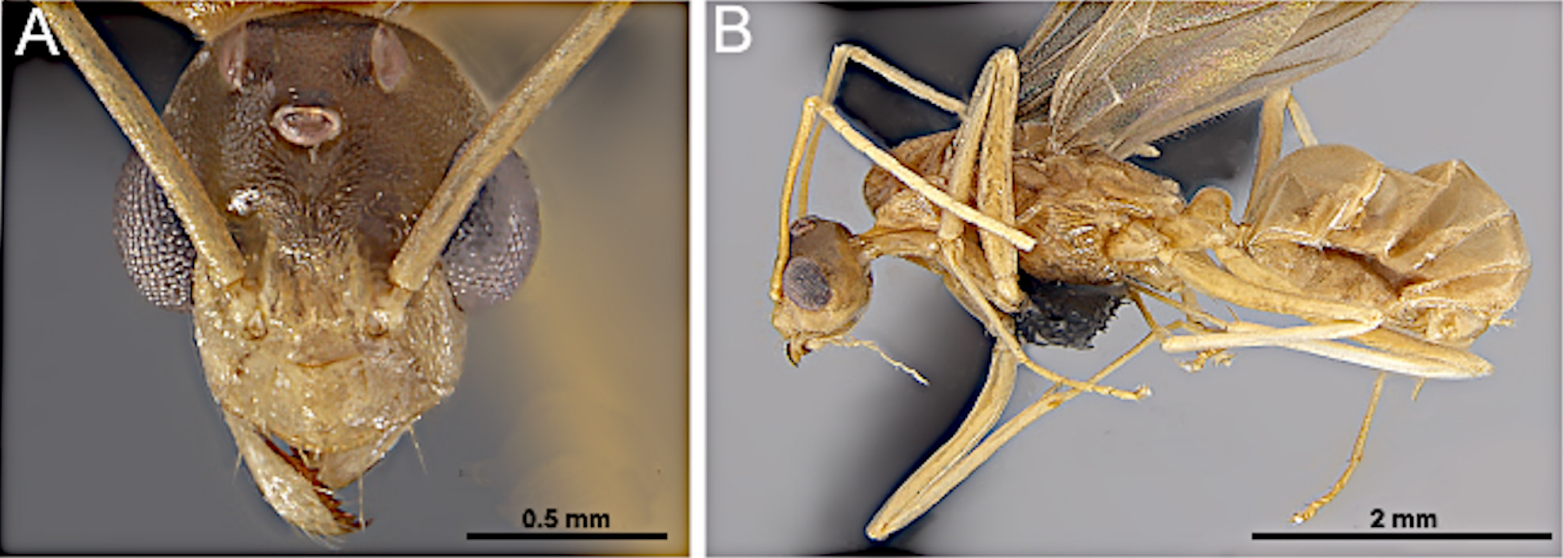
Head in full-face view (**A**) and body in lateral view (**B**) of *Anoplolepis gracilipes* (MEM 206500; photos by Joe MacGown).

Figs 6A, 6C, 36A, 36B

##### Diagnosis.

***Head*** (Fig. 36A). Antenna with 12 segments. Medium-sized, overall length 4–6 mm. Mandible well-developed, triangular; masticatory margin with eight or nine denticles. Palp formula 6:4; maxillary palp exceeding hypostomal margin but not reaching occipital foramen. Antennal sockets confluent with the posterior margin of the clypeus. Scape lacking erect macrosetae; approximately twice as long as head length. Funiculus subequal in length to mesosoma; first flagellomere slightly shorter than the second flagellomere in medial view. Frons lacking paired coarse macrosetae. Malar space with lateral margin longer than maximum scape width.

***Mesosoma*** (Fig. 36B): pronotum distinct, anterior margin slightly concave, anterior and dorsal margins of mesoscutum forming a right rounded angle; mesoscutellum flattened; propodeum protruding.

***Male genitalia***. Parameres elongate, apically rounded; volsella moderately developed; aedeagus elongate.

***Wings*** present. ***Forewing*** (Fig. 34C): pterostigma reduced; costal vein (C) present; fused subcostal and radial veins (Sc+R) present; cross vein (2r-rs) connected with radial sector vein at the midpoint of pterostigma; radial sector-medial vein (Rs+M) absent; radial sector vein (Rs) reaching the costal margin; medial-cubital (M+Cu) vein present; first medial-cubital crossvein (1m-cu) absent; crossvein (2rs-m) absent; media vein (M) incomplete, not reaching the wing margin; cubital-anal crossvein (cu-a) proximal to junction between media and cubitus; anal vein (A) longer than the fused medial-cubital vein M+Cu. ***Hindwing*** (Fig. 34D): fused radius and radial sector vein (R+Rs) present; medial-cubital (M+Cu) vein absent; fused radial sector-medial crossvein and medial vein (1rs-m+M) present; cubital-anal crossvein (cu-a) present; free section of cubitus (Cu) present; anal vein (A) absent.

##### Remarks.

Males of *Anoplolepis* are characterized by extremely elongate scape (~ 2 × head length) lacking erect macrosetae, 12-segmented antenna, and a funiculus subequal to mesosoma length. They are further distinguished by well-developed triangular mandible with numerous denticles (8 or 9), absence of paired macrosetae on the frons, and an elongate malar space. Although superficially similar to *Paratrechina* in possessing elongate scape, *Anoplolepis* differs by the absence of dorsal pilosity (present in *Paratrechina*) and by the nodiform petiolar node.

##### Biology and distribution.

In the United States, *Anoplolepis gracilipes* has been recorded from Honolulu, Hawaii (AntWeb 2025). Although there are currently no confirmed records from the continental Nearctic region, this species is a globally distributed tramp ant with strong invasive potential and may establish in suitable habitats of the southeastern United States; it is therefore included here for identification purposes.

#### Plagiolepis

Taxon classification

Animalia

HymenopteraFormicidae

Mayr, 1861

5081F048-A608-5607-B1FF-F8DB99273B89

##### Note.

In the Nearctic region, the genus *Plagiolepis* Mayr, 1861 is represented by the introduced species *Plagiolepis alluaudi* Emery, 1894 (Bolton 2025).

#### Plagiolepis alluaudi

Taxon classification

Animalia

HymenopteraFormicidae

Emery, 1894

64468B90-A560-5A5E-8375-06849B24E658

**Figure 37.**
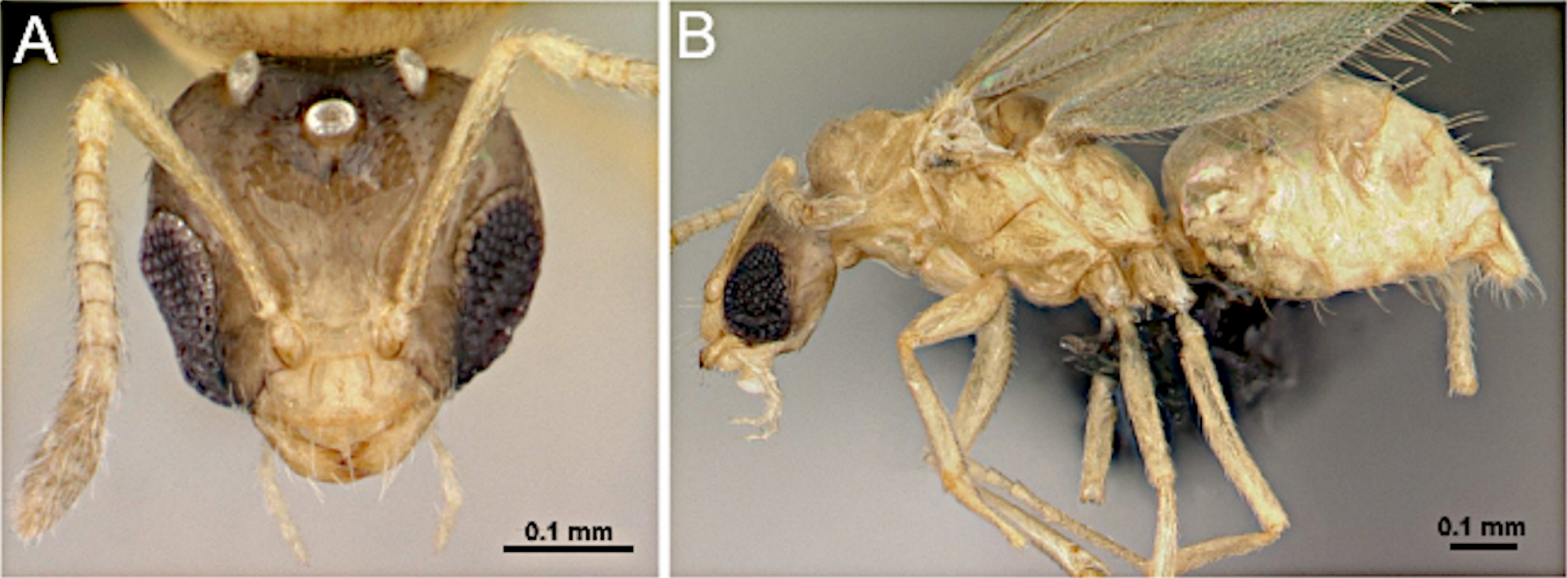
Head in full-face view (**A**) and body in lateral view (**B**) of *Plagiolepis alluaudi* (CASENT0495472; photos by Erin Prado; AntWeb.org).

Figs 5A, 6D, 6E, 7C, 7D, 8A, 8B, 37A, 37B

##### Diagnosis.

***Head*** (Fig. 37A). Antenna with 12 segments. Males minute, overall length 1.5–2.5 mm. Mandible relatively broad, subtriangular; masticatory margin with two or three teeth. Palp formula 6:4; maxillary palp slightly longer than compound eye. Antennal sockets directly contacting the posterior clypeal margin. Scape lacking erect macrosetae, slightly longer than head length. Funiculus shorter than mesosoma length; first flagellomere ~ 2 × the length of the second in medial view. Frons lacking paired coarse macrosetae. Malar space laterally compressed, shorter than scape width.

***Mesosoma*** (Fig. 37B): Dorsal margin of pronotum short and vertical; anterior margin of mesoscutum rounding into flat dorsal face; mesoscutellum weakly developed; propodeum with distinct dorsal face longer than declivitous face.

***Male genitalia***. Parameres short, apically rounded; volsella reduced; aedeagus short.

***Wings*** present. ***Forewing*** (Fig. 34E): pterostigma well developed; costal vein (C) present; fused subcostal and radial veins (Sc+R) present; cross vein (2r-rs) connected with radial sector vein at the midpoint of pterostigma; radial sector-medial vein (Rs+M) absent; radial sector vein (Rs) reaching the costal margin; medial-cubital (M+Cu) vein present; first medial-cubital crossvein (1m-cu) absent; crossvein (2rs-m) absent; media vein (M) incomplete, not reaching the wing margin; cubital-anal crossvein (cu-a) proximal to junction between media and cubitus; anal vein (A) longer than the fused medial-cubital vein M+Cu. ***Hindwing*** (Fig. 34F): fused radius and radial sector vein (R+Rs) present; medial-cubital (M+Cu) vein absent; fused radial sector-medial crossvein and medial vein (1rs-m+M) absent; cubital-anal crossvein (cu-a) absent; free section of cubitus (Cu) absent; anal vein (A) absent.

##### Remarks.

Males of *Plagiolepis* in the Nearctic region are minute ants recognized by the following characters: antenna 12-segmented, scape lacking erect macrosetae, malar space relatively short, and the very small body size. They may resemble small males of *Brachymyrmex*, but differ in the higher antennal segmentation and the distinctive wing venation, notably the absence of the forewing crossvein 2rs–m and the strongly reduced hindwing venation. These characters easily distinguish the genus from other Nearctic formicine genera.

##### Biology and distribution.

In the Nearctic region, *Plagiolepis alluaudi* has been recorded from Florida (Chouvenc et al. 2018). The species is introduced and appears to occur primarily in disturbed habitats associated with human activity. Colonies typically nest in soil, leaf litter, or other sheltered microhabitats in warm environments.

## Discussion

The male-based key to Nearctic Formicinae genera presented here complements existing identification resources, which have focused primarily on workers and queens. Although males are frequently collected during ecological surveys, they have long been underrepresented in taxonomic treatments because of limited diagnostic characters and reduced morphological differentiation. By incorporating characters of genitalia, antennal segmentation, mandible and mesosoma structure, and wing venation, this key enables more consistent and accurate identification of males to the genus level. High-resolution imaging and updated comparative matrices further enhance its utility for taxonomists and biodiversity monitoring efforts.

Wingless males in *Nylanderia* represent a notable departure from the typical formicine condition, in which males are fully winged and adapted for dispersal. These males are associated with in-nest mating strategies, likely reducing dispersal risks while promoting localized gene flow. They commonly exhibit reductions in thoracic sclerites and flight musculature, along with modifications of the antenna. However, their rarity and frequent resemblance to workers have contributed to persistent taxonomic challenges. In *Nylanderia*, this condition is linked to socially parasitic life histories, with apterous males documented in *N. deceptrix*, *N. deyrupi*, and *N. parasitica* (Messer et al. 2016; Messer et al. 2020).

Identifying species based solely on male characters remains challenging, particularly in diverse genera where male morphology is conserved, such as *Camponotus* and *Lasius*. Ongoing efforts to associate males with workers and queens through nest collections, rearing, and molecular barcoding will be critical for refining identifications and resolving taxonomic ambiguities. Overall, this key underscore the importance of male morphology in ant systematics and highlights its potential to improve the understanding of Nearctic Formicinae diversity and evolution.

## Supplementary Material

XML Treatment for Camponotus

XML Treatment for Colobopsis

XML Treatment for Formica

XML Treatment for Polyergus

XML Treatment for Lasius

XML Treatment for Myrmecocystus

XML Treatment for Nylanderia

XML Treatment for Paratrechina

XML Treatment for Paratrechina longicornis

XML Treatment for Prenolepis

XML Treatment for Prenolepis imparis

XML Treatment for Brachymyrmex

XML Treatment for Myrmelachista

XML Treatment for Acropyga

XML Treatment for Acropyga epedana

XML Treatment for Anoplolepis

XML Treatment for Anoplolepis gracilipes

XML Treatment for Plagiolepis

XML Treatment for Plagiolepis alluaudi

## Additional information

### Artificial Intelligence (AI) use

The authors accept full responsibility for the content of the manuscript, including the disclosure of any use of AI.

No AI tools were used in the preparation of this manuscript.

### Author ORCIDs

N. Rasoamanana https://orcid.org/0000-0001-9702-7231

J.A. MacGown https://orcid.org/0009-0005-2861-0593

B.L. Fisher https://orcid.org/0000-0002-4653-3270

J.G. Hill https://orcid.org/0000-0002-1892-7117

### Data availability

All of the data that support the findings of this study are available in the main text or Supplementary Information.

## Supplementary materials

Supplementary material 1 Examined material Data type xls Explanation note Detailed collection information and unique identifiers for examined specimens. https://binary.pensoft.net/file/1650943This dataset is made available under the Open Database License (http://opendatacommons.org/licenses/odbl/1.0/). The Open Database License (ODbL) is a license agreement intended to allow users to freely share, modify, and use this Dataset while maintaining this same freedom for others, provided that the original source and author(s) are credited. Nicole Rasoamanana, Joe A. MacGown, Brian L. Fisher, JoVonn G. Hill

## References

[B1] AntWeb (2025) AntWeb, California Academy of Science, San Francisco, California, USA. http://www.antweb.org [accessed 19 September 2025]

[B2] Blaimer BB, Brady SG, Schultz TR, Lloyd MW, Fisher BL, Ward PS (2015) Phylogenomic methods outperform traditional multi-locus approaches in resolving deep evolutionary history: a case study of formicine ants. BMC Evolutionary Biology 15: 271. 10.1186/s12862-015-0552-5 PMC4670518 26637372

[B3] Bolton B (2025) An online catalog of the ants of the world. https://antcat.org [accessed 19 September 2025]

[B4] Boudinot BE (2013) The male genitalia of ants: musculature, homology, and functional morphology (Hymenoptera, Aculeata, Formicidae). Journal of Hymenoptera Research 30: 29–49. 10.3897/jhr.30.3535

[B5] Boudinot BE (2015) Contributions to the knowledge of Formicidae (Hymenoptera, Aculeata): A new diagnosis of the family, the first global male-based key to subfamilies, and a treatment of early branching lineages. European Journal of Taxonomy 120(120): 1–62. 10.5852/ejt.2015.120

[B6] Boudinot BE, Fisher BL (2013) A taxonomic revision of the *Meranoplus* F. Smith of Madagascar (Hymenoptera: Formicidae: Myrmicinae) with keys to species and diagnosis of the males. Zootaxa 3635: 301–339. 10.11646/zootaxa.3635.4.1 26097952

[B7] Cantone S (2017) Winged Ants, the Male. Dichotomous Key to Genera of Winged ♂♂ Ants in the World; Behavioral Ecology of Mating Flight. Stefano Cantone, Catania, Italy, 318 pp. [ISBN-A 10.979.12200/23948]

[B8] Chouvenc T, Scheffrahn RH, Warner J (2018) Establishment of Alluaud’s little yellow ant, *Plagiolepis alluaudi* Emery (Hymenoptera: Formicidae: Formicinae): first continental New World record. Florida Entomologist 101: 138–140. 10.1653/024.101.0126

[B9] Deyrup M (2017) Ants of Florida: Identification and Natural History. CRC Press, Boca Raton, FL, 423 pp.

[B10] Deyrup M, Davis L, Cover S (2000) Exotic ants in Florida. Transactions of the American Entomological Society 126: 293–326.

[B11] LaPolla JS (2004) *Acropyga* (Hymenoptera: Formicidae) of the world. Contributions of the American Entomological Institute 33(3): 1–130.

[B12] LaPolla JS, Cover SP, Mueller UG (2002) Natural history of the mealybug-tending ant *Acropyga epedana*, with descriptions of the male and queen castes. Transactions of the American Entomological Society 128(3): 367–376.

[B13] Messer SJ, Cover SP, LaPolla JS (2016) *Nylanderia deceptrix* sp. n., a new species of obligately socially parasitic formicine ant (Hymenoptera, Formicidae). ZooKeys 552: 49–65. 10.3897/zookeys.552.6475 PMC4740849 26865815

[B14] Messer SJ, Cover SP, Rabeling C (2020) Two new species of socially parasitic *Nylanderia* ants from the southeastern United States. ZooKeys 921: 23–48. 10.3897/zookeys.921.46921 PMC7109158 32256149

[B15] Ramamonjisoa MM, Rasoamanana N, Fisher BL (2024) Male-based key to the subfamilies and genera of Malagasy ants (Hymenoptera, Formicidae). ZooKeys 1213: 289–259. 10.3897/zookeys.1213.120531 PMC11452741 39372281

[B16] Smith MR (1943) A generic and subgeneric synopsis of the male ants of the United States. American Midland Naturalist 30: 273–321. 10.2307/2421283

[B17] Ward PS, Boudinot BE (2021) Grappling with homoplasy: taxonomic refinements and reassignments in the ant genera *Camponotus* and *Colobopsis* (Hymenoptera: Formicidae). Arthropod Systematics & Phylogeny 79: 37–56. 10.3897/asp.79.e66978

[B18] Williams JL, Punnath A, Fernández MB, Calcaterra LA, LaPolla JS, Lucky A (2024) “Picking up signals” in male genital morphospace and integrating phylogenomics to delimit Neotropical *Nylanderia* Emery species (Hymenoptera: Formicidae). Insect Systematics and Diversity 8(5): 6. 10.1093/isd/ixae028

[B19] Wilson EO (1955) A monographic revision of the ant genus *Lasius*. Bulletin of the Museum of Comparative Zoology 113: 1–201.

[B20] Yoshimura M, Fisher BL (2007) A revision of male ants of the Malagasy region (Hymenoptera: Formicidae): Key to subfamilies and treatment of the genera of Ponerinae. Zootaxa 1654(1): 21–40. 10.11646/zootaxa.1654.1.2

[B21] Yoshimura M, Fisher BL (2009) A revision of male ants of the Malagasy region (Hymenoptera: Formicidae): Key to genera of the subfamily Proceratiinae. Zootaxa 2216: 1–21. 10.11646/zootaxa.2216.1.1

